# Downregulation of histone H2A and H2B pathways is associated with anthracycline sensitivity in breast cancer

**DOI:** 10.1186/s13058-016-0676-6

**Published:** 2016-02-06

**Authors:** Marsela Braunstein, Linda Liao, Nicola Lyttle, Nazleen Lobo, Karen J. Taylor, Paul M. Krzyzanowski, Irina Kalatskaya, Cindy Q. Yao, Lincoln D. Stein, Paul C. Boutros, Christopher J. Twelves, Richard Marcellus, John M. S. Bartlett, Melanie Spears

**Affiliations:** Ontario Institute for Cancer Research, MaRS Centre, 661 University Avenue, Toronto, ON M5G 0A3 Canada; Department of Immunology, University of Toronto, Toronto, ON Canada; Edinburgh Cancer Research Centre, Western General Hospital, Edinburgh, UK; Department of Molecular Genetics, University of Toronto, Toronto, ON Canada; Department of Medical Biophysics, University of Toronto, Toronto, ON Canada; Department of Pharmacology & Toxicology, University of Toronto, Toronto, Canada; Leeds Institute of Cancer and Pathology and Cancer Research UK Centre, St James’s University Hospital, Leeds, UK; Department of Laboratory Medicine and Pathobiology, University of Toronto, Toronto, ON Canada

**Keywords:** Breast cancer, Anthracycline resistance, Gene expression, Small-molecule inhibitors, Clinical trial, Histone

## Abstract

**Background:**

Drug resistance in breast cancer is the major obstacle to effective treatment with chemotherapy. While upregulation of multidrug resistance genes is an important component of drug resistance mechanisms in vitro, their clinical relevance remains to be determined. Therefore, identifying pathways that could be targeted in the clinic to eliminate anthracycline-resistant breast cancer remains a major challenge.

**Methods:**

We generated paired native and epirubicin-resistant MDA-MB-231, MCF7, SKBR3 and ZR-75-1 epirubicin-resistant breast cancer cell lines to identify pathways contributing to anthracycline resistance. Native cell lines were exposed to increasing concentrations of epirubicin until resistant cells were generated. To identify mechanisms driving epirubicin resistance, we used a complementary approach including gene expression analyses to identify molecular pathways involved in resistance, and small-molecule inhibitors to reverse resistance. In addition, we tested its clinical relevance in a BR9601 adjuvant clinical trial.

**Results:**

Characterisation of epirubicin-resistant cells revealed that they were cross-resistant to doxorubicin and SN-38 and had alterations in apoptosis and cell-cycle profiles. Gene expression analysis identified deregulation of histone H2A and H2B genes in all four cell lines. Histone deacetylase small-molecule inhibitors reversed resistance and were cytotoxic for epirubicin-resistant cell lines, confirming that histone pathways are associated with epirubicin resistance. Gene expression of a novel 18-gene histone pathway module analysis of the BR9601 adjuvant clinical trial revealed that patients with low expression of the 18-gene histone module benefited from anthracycline treatment more than those with high expression (hazard ratio 0.35, 95 % confidence interval 0.13–0.96, *p* = 0.042).

**Conclusions:**

This study revealed a key pathway that contributes to anthracycline resistance and established model systems for investigating drug resistance in all four major breast cancer subtypes. As the histone modification can be targeted with small-molecule inhibitors, it represents a possible means of reversing clinical anthracycline resistance.

**Trial registration:**

ClinicalTrials.gov identifier NCT00003012. Registered on 1 November 1999.

**Electronic supplementary material:**

The online version of this article (doi:10.1186/s13058-016-0676-6) contains supplementary material, which is available to authorized users.

## Background

Breast cancer is the second leading cause of cancer death for women. Most patients present with early disease and are treated with surgery, often followed by adjuvant radiotherapy and chemotherapy with or without endocrine therapy or trastuzumab given with curative intent. Nevertheless, 40–50 % of high-risk patients treated with adjuvant chemotherapy ultimately relapse as a result of having resistant disease [1]. Despite the advent of targeted therapies, chemotherapy is also central to the treatment of women with metastatic disease, who often respond to palliative chemotherapy but in due course relapse due to drug resistance, including cross-resistance to structurally unrelated anti-cancer drugs [2].

The taxanes and anthracyclines are widely used as adjuvant therapy as well as in metastatic cancer. Both target rapidly proliferating cancer cells. The taxanes interfere with microtubule depolymerisation, causing cell-cycle arrest [3, 4], whereas anthracyclines introduce DNA breaks, form free radicals and covalently bind type II topoisomerase (Topo II)–DNA complexes [5]. The taxanes and anthracyclines are both natural products and susceptible to resistance mediated by over-expression of the multidrug transporter P-glycoprotein. A well-established in vitro mechanism of resistance involves activity of multidrug resistance genes 1 and 2/3 (*MDR1* and *MDR2/3*, respectively), which bind non-specifically to multiple drugs and actively export them across the cellular membrane [6, 7]. Although this results in decreased intra-cellular drug concentrations and cytotoxicity, the clinical relevance of MDR genes remains to be determined. Other mechanisms include reduced Topo activity [8, 9], reduced Fas ligand expression [10] and downregulation of *TP53* expression [11]. However, the molecular drivers of clinical anthracycline resistance remain largely unknown. We previously identified duplication of centromeric region on chromosome 17 (CEP17), a surrogate marker of chromosomal instability, as a predictive marker of clinical anthracycline sensitivity [12–14]. However, identifying pathways that could be targeted in the clinic to eliminate anthracycline-resistant breast cancer remains a major challenge.

The aim of this study was to establish anthracycline-resistant breast cancer cell lines to (1) identify pathways driving resistance that are common to all breast cancers, regardless of their oestrogen receptor (ER) and human epidermal growth factor receptor 2 (HER2) status; (2) discover a predictive biomarker of anthracycline benefit; and (3) investigate alternative treatment options for patient groups that are not expected to respond to anthracycline regimens. Cell lines were chosen to reflect four major breast cancer subtypes [15, 16]: MCF7 (ER+/HER2−, luminal A), ZR-75-1 (ER+/HER2+, luminal B), SKBR3 (ER−/HER2+, HER2-amplified) and MDA-MB-231 (ER−/progesterone receptor–negative [PR−]/HER2−, triple-negative), and they were exposed to increasing concentrations of epirubicin until resistant cells were generated. To identify mechanisms driving epirubicin resistance, we used complementary approaches, including gene expression analyses to identify signalling pathways involved in resistance and small-molecule inhibitors to reverse resistance. We demonstrated that a histone H2A- and H2B-containing module was associated with epirubicin resistance and that small-molecule inhibitors targeting histone pathways induced cytotoxicity in all epirubicin-resistant cell lines. Most importantly, the identified mechanism of resistance was recapitulated in the BR9601 clinical trial, where the patients with low expression of the histone module benefited from anthracycline treatment compared with patients with high expression of the same module (hazard ratio [HR] 0.35, 95 % confidence interval [CI] 0.13–0.96, *p* = 0.042). Thus, in our study, we identified that chromatin remodelling represents an important mechanism of anthracycline resistance in breast cancer and established a reliable in vitro model system for investigating anthracycline resistance in all four breast cancer subtypes. As the histone modification can be targeted with small-molecule inhibitors, it presents a possible means of reversing clinical anthracycline resistance.

## Methods

### BR9601 trial

The BR9601 trial (ClinicalTrials.gov identifier NCT0003012) investigators recruited 374 pre- and post-menopausal women with completely excised, histologically confirmed breast tumours and a clear indication for adjuvant chemotherapy. Patients were randomised between 8 cycles of CMF (intravenous cyclophosphamide 750 mg/m^2^, methotrexate 50 mg/m^2^ and 5-fluorouracil 600 mg/m^2^) every 21 days, and E-CMF (4 cycles of epirubicin 100 mg/m^2^ every 21 days followed by 4 cycles of the same CMF regimen) [17] (Additional file 1: Figure S1). The protocol was approved by central and local ethics committees, and each patient provided written informed consent before randomisation. For the present analysis, tissue blocks were retrieved and RNA was extracted. The primary outcomes of the BR9601 study were relapse-free survival and OS, although distant relapse-free survival (DRFS) was also reported [17].

### Cell culture

Breast cancer cell lines (MDA-MB-231, MCF7, ZR-75-1, SKBR3) were purchased from the American Type Culture Collection (Manassas, VA, USA) and cultured in Dulbecco’s modified Eagle’s medium (except SKBR3, cultured in RPMI 1640 medium) supplemented with 10 % heat-inactivated foetal bovine serum and Gibco 1 % l-glutamine (Thermo Scientific, Burlington, ON, Canada). Epirubicin-resistant cell lines were generated by exposing native cells to increasing concentrations of epirubicin with an initial concentration set at 0.5 nM. Resistance was defined when the half-maximal inhibitory concentration (IC_50_) value superseded the IC_50_ value of the corresponding native cell line and resistant cells could not tolerate further increase in drug concentration. Drug resistance and cross-resistance were determined by exposing cells to drug concentrations ranging from 0.3 to 3000 nM for 72 h. Cell viability was determined using the Cell Counting Kit-8 (CCK-8; Dojindo Molecular Technologies/Cedarlane Laboratories, Burlington, ON, Canada). IC_50_ values were calculated using Prism 5 software (GraphPad Software, La Jolla, CA, USA).

### Flow cytometry

For cell-cycle analysis, cells were synchronised by the double-thymidine block [18] and incubated with dimethyl sulphoxide (DMSO) or epirubicin doses established for each cell line: 25 nM for MDA-MB-231, 30 nM for MCF7, 15 nM for SKBR3 and 10 nM for ZR-75-1. Cells were collected at 48 h, fixed with 80 % ethanol and incubated with 2 mg/ml RNase A and 0.1 mg/ml propidium iodide (Sigma-Aldrich, Oakville, ON, Canada) before analysis. For apoptosis experiments, cells were treated with DMSO or epirubicin at the concentrations described above and collected at 72 h for staining with annexin V apoptosis detection eFluor 450 (eBioscience, San Diego, CA, USA). Data were collected using a FACSCanto II flow cytometer and FACSDiva software (BD Biosciences, Mississauga, ON, Canada) and analysed using FlowJo software (Treestar, Ashland, OR, USA).

### Cell proliferation

Cells were cultured in the presence or absence of epirubicin for up to 96 h (see Flow cytometry section above for epirubicin concentrations). Cells were collected at 24, 48, 72 and 96 h and counted using a Vi-CELL Cell Viability Analyzer (Beckman Coulter, Mississauga, ON, Canada). Data were analysed using GraphPad Prism 5 software.

### Microarray

Illumina HumanHT-12 v4 BeadChips (Illumina, San Diego, CA, USA) were used for the whole genome microarray analysis by the UHN Microarray Centre, Toronto, ON, Canada. Total RNA was extracted with the RNeasy Mini Kit (Qiagen, Toronto, ON, Canada) and used for profiling gene expression changes. Raw data (Gene Expression Omnibus accession number [GEO:GSE54326]) were normalised with the R3.0.0 lumi package using simple scaling normalisation; the 10 % most variable probes were retained for differential analysis using the genefilter package. Differentially expressed probes were identified using limma with a Benjamini–Hochberg corrected *p* value cut-off of 0.05.

### Network-based analysis

To identify functionally relevant modules, genes demonstrating consistent directionality of significant expression changes were analysed using the Cytoscape Reactome Functional Interaction (FI) plugin in Cytoscape 2.8.3. Symbols were loaded as a gene set and interactions from the FI network 2012 version, including FI annotations and linker genes. Network modules were identified using spectral clustering and pathway enrichment computed for each module using the Reactome FI plugin functions. Reactome pathways exhibiting false discovery rate (FDR) values less than 0.01 were considered enriched.

### Pharmaceutical inhibitors

All inhibitors were provided by the drug discovery group at the Ontario Institute for Cancer Research (Toronto, ON, Canada). Cells were seeded at 1000–1500 cells/well into 384-well plates (Greiner Bio-One, Mississauga, ON, Canada). After 24 h, resistant cells were exposed to epirubicin at the selection doses established (see Flow cytometry section above), then exposed to histone deacetylase (HDAC) inhibitors (HDACi) dissolved in DMSO in 12 concentrations ranging from 0.0026 to 10 μM using HP D300 digital compound dispenser (Tecan Systems, San Jose, CA, USA). The DMSO concentration did not exceed 0.5 % in the final drug solution. After 72 h, the effects of inhibitors were determined using CellTiter-Glo Luminescent Cell Viability Assay (Promega, Madison, WI, USA) and the Wallac EnVision 2104 Multilabel Reader (PerkinElmer, Woodbridge, ON, Canada). Raw data were normalised to negative (media) and positive (20 μM staurosporine) controls and analysed using GraphPad Prism 5.

### Quantitative RT-PCR

RNA was isolated from cultured cell lines using the RNeasy Mini Kit (Qiagen, Toronto, ON, Canada). A total of 20 ng of RNA was analysed using TaqMan gene expression assays (HIST1H2BD, Hs00371070_m1; HIST1H2BK, Hs00955067_g1; HIST1H2AC, Hs00185909_m1) and EXPRESS One-Step Superscript qRT-PCR universal kit according to manufacturer’s protocol (Life Technologies, Burlington, ON, Canada). Reactions were run using Applied Biosystems ViiA 7 Real-Time PCR instrument and software (Life Technologies). Transcript levels were quantified from the standard curve generated from the control Universal Human Reference RNA samples (Agilent, Mississauga, ON, Canada). Statistical significance was determined using an unpaired *t* test.

### Immunoblotting

Whole-cell lysates (WCL) were prepared in radioimmunoprecipitation assay (RIPA) buffer supplemented with cOmplete Mini Protease and PhosSTOP phosphatase inhibitors (Roche, Laval, QC, Canada). For cell line characterisation, 10–50 μg of total protein was run on 4–20 % Mini-PROTEAN TGX Precast Gels (Bio-Rad Laboratories, Mississauga, ON, Canada). For histones, cells were collected in 0.1 % Nonidet P-40 in phosphate-buffered saline to release nuclei. WCL were prepared by adding equal volumes of 2× RIPA buffer supplemented with 25 U of Benzonase Nuclease (Sigma-Aldrich) and cOmplete Mini Protease Inhibitor Cocktail (Roche), incubating on ice for 30 minutes and sonicating for 15 minutes with 30-second on-off intervals. Twenty micrograms of WCL were run on a 12 % gel. A list of primary antibodies used in immunoblotting is provided in Additional file 1: Table S5. Signals were developed with BM Chemiluminescence Blotting Substrate (POD) (Roche) and a ChemiDoc imaging system (Bio-Rad Laboratories).

### Small interfering RNA transfection of ZR75-1- and MDA-MB-231-resistant cells

For CCK-8 assays, a total of 7 × 10^4^ ZR75-1 epirubicin-resistant cells and MDA-MB-231 epirubicin-resistant cells were transfected with Lipofectamine RNAiMAX (Life Technologies) and 10 nM Dharmacon ON-TARGET*plus* siRNA reagent human SMARTpool (GE Healthcare, Lafayette, CO, USA) targeting HIST1H2AC (L-011435-01-0005), HIST1H2BK (L-013323-02-0005) or both according to the manufacturer’s instructions. Negative controls included media only, Lipofectamine only or mock transfection with non-targeting small interfering RNA (siRNA; D-001810-10-05). Cells were exposed to 0.3–3000 nM epirubicin for 72 h before their viability was determined using the CCK-8 kit. For flow cytometric analyses, 2 × 10^5^ cells were plated in 6-well plates and transfected with 10 nM siRNA or control as described above. Samples were collected at 72 h for quantitative real-time polymerase chain reaction (qRT-PCR) and flow cytometric analyses.

### nCounter CodeSet and data pre-processing

The nCounter gene expression CodeSet (NanoString Technologies, Seattle, WA, USA) included 7 genes within the histone module and 11 additional genes that were identified in the KEGG PATHWAY database [19] as being important for histone function (Additional file 1: Table S6). *HIST1H2AC* was excluded from the CodeSet because probes cross-hybridised to other genes. All 18 genes were functionally related (Additional file 1: Figure S6). Messenger RNA (mRNA) CodeSets were processed on nCounter according to the manufacturer’s instructions. Raw mRNA abundance data were pre-processed using the NanoStringNorm R package (v1.1.19; Additional file 2: Methods). A range of pre-processing schemes was assessed to optimise normalisation parameters as previously described (Haider S., Yao C. Q., Sabine V. S., Grzadkowski M., Starmans M. H. W., Wang J., Nguyen F., Moon N. C., Lin X., Drake C., Crozier C. A., Brookes C. L., van de Velde C. J. H., Hasenburg A., Kieback D. G., Markopoulos C. J., Dirix L. Y., Seynaeve C., Rea D. W., Kasprzyk A., Lambin P., Lio P., Bartlett J. M. S., Boutros P. C.: Using pathways for cross-disease biomarker discovery, in preparation).

### Survival modelling

To assess whether individual genes are prognostic of survival, each gene was median dichotomised into low- and high-expression groups and univariate Cox proportional hazards models were fit (Additional file 1: Figure S7). Survival analysis of clinical variables modelled age as binary variable (dichotomised at age >50 years), while nodal status, pathological grade, ER status and HER2 status were modelled as ordinal variables (Additional file 1: Figure S1B). Tumour size was treated as a continuous variable.

### mRNA network analysis

We hypothesised that integrating molecular modules could improve residual risk prediction relative to DRFS and OS. For each module, we calculated a module dysregulation score (Additional file 2: Methods), which we used in a univariate Cox proportional hazards model. A stratified fivefold cross-validation approach was applied, and models were trained in the training cohort and validated in the *k*th testing cohort using 10-year DRFS as an end point. All survival modelling was performed on DRFS and OS in the R statistical environment with the survival package (v2.37-7). Treatment by marker interaction term was calculated using Cox proportional hazards model.

## Results

### Generation and characterisation of epirubicin-resistant breast cancer cell lines

**Fig. 1:**
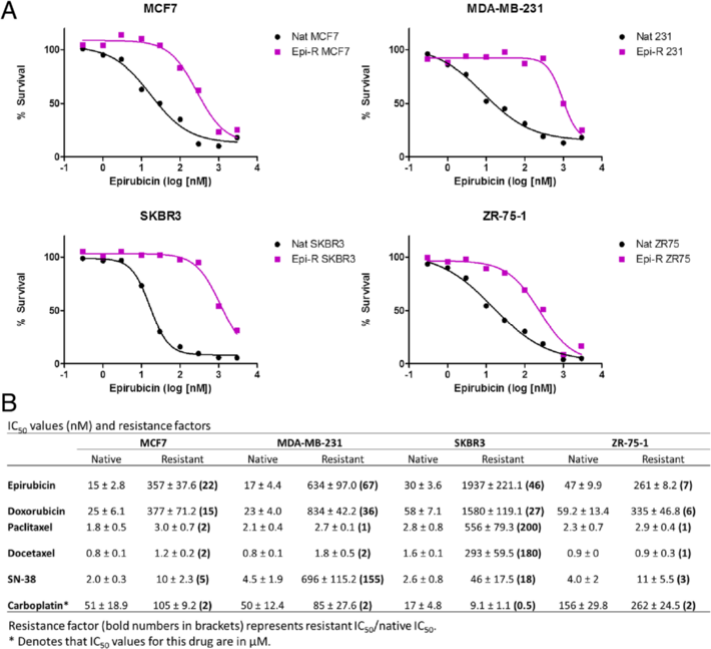
Characterisation of epirubicin-resistant cell lines. Native and resistant cells were exposed to drug concentrations ranging from 0.3 to 3000 nM. Cell viability was determined 72 h later by Cell Counting Kit-8 assay. **a** Percentage of live cells relative to dimethyl sulphoxide control was plotted against epirubicin concentration. *Black* = native cells, *magenta* = resistant cells. **b** Half-maximal inhibitory concentration (IC_50_) values in nanomolar concentrations ± standard deviation. Resistance factor is shown in parentheses and represents resistant IC_50_/native IC_50_

Resistant cell lines generated from epirubicin-sensitive native cell lines MDA-MB-231, MCF7, SKBR3 and ZR-75-1 exhibited 7- to 67-fold increased resistance to epirubicin (Fig. 1). We tested whether epirubicin-resistant cell lines are cross-resistant to doxorubicin, paclitaxel, docetaxel, SN-38 and carboplatin, drugs used in breast cancer clinical trials. All four epirubicin-resistant cell lines were resistant to doxorubicin (Fig. 1b). While MDA-MB-231, MCF7 and ZR-75-1 epirubicin-resistant cells were not taxane-resistant, SKBR3 epirubicin-resistant cells were cross-resistant to both paclitaxel and docetaxel (Fig. 1b). MDA-MB-231 and SKBR3 cells were cross-resistant to SN-38, whereas MCF7 and ZR-75-1 tolerated only small increases in SN-38 concentrations. None of the cell lines were cross-resistant to carboplatin (Fig. 1b).

**Fig. 2:**
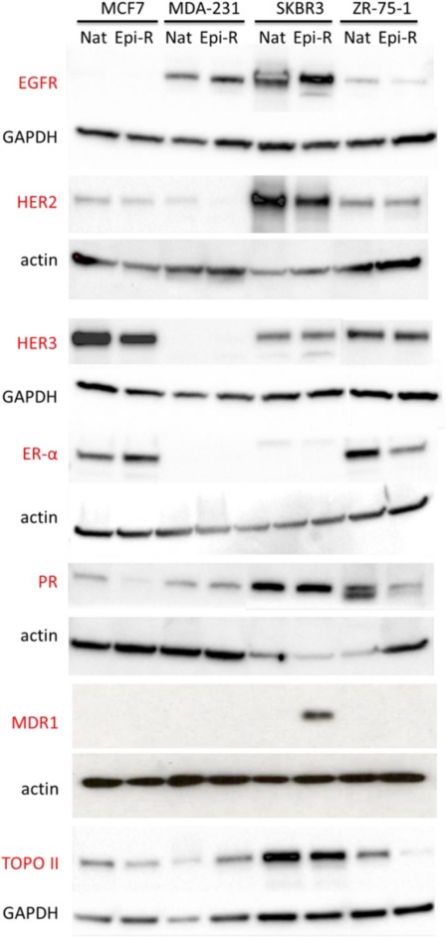
Expression of conventional breast cancer biomarkers and selected multidrug resistance genes. Cell lysates were prepared in radioimmunoprecipitation assay buffer supplemented with cOmplete Mini protease inhibitor and PhosSTOP phosphatase inhibitor. Quantities (10–50 μg) of total protein were run on a 10 % gel (MDR1), 4–20 % precast gels (epidermal growth factor receptor [EGFR], oestrogen receptor [ER], progesterone receptor [PR], type II topoisomerase [TOPO IIα]) and Any kD Mini-PROTEAN TGX Precast Protein Gels (human epidermal growth factor receptor 2 [HER2], HER3), transferred onto polyvinylidene fluoride membrane and developed using chemiluminescence substrate. *Nat* native, *Epi-R* epirubicin-resistant, *GAPDH* glyceraldehyde-3-phosphate dehydrogenase

Epirubicin-resistant cells showed slightly decreased levels of ER and PR in ZR-75-1 cells compared with native cells. The levels of epidermal growth factor receptor, HER2 and HER3 also remained unchanged (Fig. 2). Interestingly, we observed small amounts of HER2 protein in MCF7 cell lines, which was not evident by flow cytometry (Additional file 1: Figure S8), in line with classifying this cell line as being HER2− [20]. MDR1 was upregulated only in resistant SKBR3 cells, which may explain their cross-resistance to taxanes (Fig. 1b). Topo IIα expression was downregulated in epirubicin-resistant ZR-75-1 cells (Fig. 2). No changes in MDR or Topo IIα were observed in epirubicin-resistant MDA-MB-231 and MCF7 cell lines. These results suggest that anthracycline resistance is not MDR-driven for three of four cell lines and that epirubicin-resistant cell lines remained unaltered with respect to the expression of conventional breast cancer biomarkers.

To determine cell-doubling time, we cultured cells with or without epirubicin for up to 96 h. In the absence of epirubicin, the native MDA-MB-231 and MCF7 cell populations doubled every 25 h and 29 h, respectively (Additional file 1: Table S1), whereas native SKBR3 and ZR-75-1 cells grew more slowly, doubling every 45 h and 50 h, respectively. In the presence of epirubicin, doubling time increased 2.8-fold for MDA-MB-231 (*p* = 0.0371), 2.5-fold for MCF7 (non-significant), 1.3-fold for SKBR3 (*p* = 0.0494) and 1.9-fold for ZR-75-1 (*p* = 0.0258) for native cells. In contrast to the native cell lines, there were no marked changes in the doubling time of the resistant cells, regardless of whether epirubicin was added (Additional file 1: Table S1). Interestingly, in the absence of epirubicin, none of the resistant cells proliferated as rapidly as native cells, indicating that epirubicin selection induced permanent changes in resistant cells.

### Impaired apoptosis in anthracycline-resistant cells

To assess the effects of epirubicin on apoptosis, apoptotic cells were scored by flow cytometry after 72 h of exposure to epirubicin. The apoptotic index was consistently lower for resistant cells than for native controls (Table 1). In particular, MDA-MB-231 and SKBR3 epirubicin-resistant cells required a substantially higher concentration of epirubicin (1000 nM) to induce apoptosis. Even at this concentration of epirubicin, the apoptotic index was still nearly 50 % less than for the native cells (Table 1).

**Table 1:** Percentages of apoptotic cells following a 72-h epirubicin treatment

Cell line	Control	Epirubicin concentration		
**MDA-MB-231**	**DMSO**	**1 nM**	**25 nM**	**1000 nM**
Native	18 %	17 %	41 %	94 %
25 nM-R	10 %	10 %	8 %	50 %
**MCF7**	**DMSO**	**1 nM**	**30 nM**	**1000 nM**
Native	32 %	29 %	49 %	77 %
30 nM-R	20 %	24 %	23 %	78 %
**SKBR3**	**DMSO**	**1 nM**	**15 nM**	**1000 nM**
Native	22 %	26 %	24 %	59 %
15 nM-R	18 %	17 %	17 %	34 %
**ZR-72-1**	**DMSO**	**1 nM**	**10 nM**	**1000 nM**
Native	36 %	44 %	47 %	71 %
10 nM-R	29 %	28 %	29 %	62 %

*DMSO* dimethyl sulphoxide; DRFS, distant relapse-free survival

Apoptotic cells = annexin V–positive. Debris and necrotic cells (annexin V–negative, 7-aminoactinomycin D–positive) were gated out. Percentages reported are derived from a single experiment; at least two independent experiments were done for each cell line

Bold data denotes the different concentrations of epirubicin used for each cell line

### Resistant cell lines overcome epirubicin-induced G_2_/M arrest

**Fig. 3:**
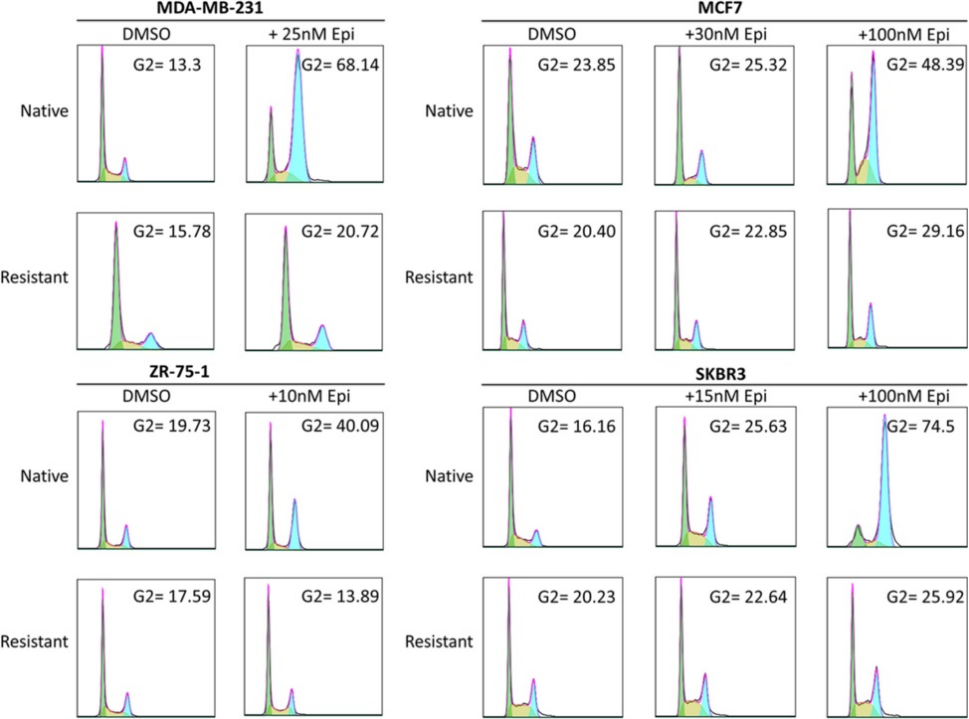
Resistant cell lines overcome epirubicin-induced G_2_/M arrest. **a**–**d** Cells were synchronised by a double-thymidine block and treated with dimethyl sulphoxide or epirubicin at selection doses established for each resistant cell line: 25 nM epirubicin to MDA-MB-231, 30 nM epirubicin to MCF7, 15 nM epirubicin to SKBR3 and 15 nM epirubicin to ZR-75-1. Epirubicin concentration was increased to 100 nM for MCF7 and SKBR3 cells because G_2_/M block was not observed at the lower doses of epirubicin. Cells were collected at 48 h, stained with propidium iodide and analysed by flow cytometry. Debris was gated out. *GAPDH* glyceraldehyde-3-phosphate dehydrogenase

Cells were synchronised before exposure to DMSO or epirubicin. All DMSO-treated cell lines progressed through the cell cycle (Fig. 3). When 25 nM and 10 nM epirubicin were added to the MDA-MB-231 and ZR-75-1 cell lines, respectively, native cells arrested in G_2_/M phase, whereas resistant cells progressed through (Fig. 3a, c). When 30 nM and 15 nM epirubicin were added to the MCF7 and SKBR3 cell lines, respectively, we observed only a modest effect on the cell cycle (Fig. 3b, d). This necessitated increasing epirubicin concentrations to 100 nM, at which level native cells arrested in G_2_/M phase but with minimal effect on the epirubicin-resistant cells (Fig. 3b, d). Therefore, overcoming a G_2_/M block may be part of the process leading to epirubicin resistance.

### Gene expression analyses identify histone H2A- and H2B-containing pathways as potential functional drivers of epirubicin resistance

**Fig. 4:**
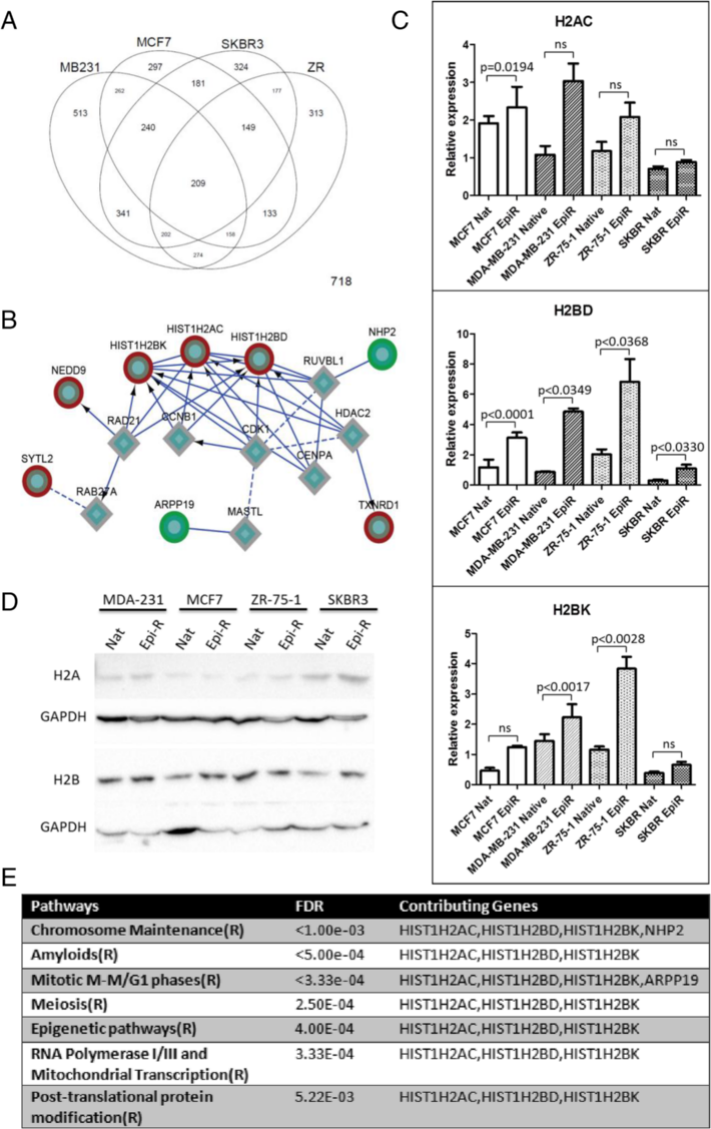
Network-based analysis of epirubicin-resistant (EpiR) cell lines. **a** Venn diagram of genes with significant changes in expression in breast cancer cell lines. **b** Histone module identified in Functional Interaction network analysis. *Coloured rings* denote genes demonstrating consistent changes across all four cell lines. *Red rings* = upregulated genes, *green rings* = downregulated genes, *diamonds* = linker genes. **c** Quantitative real-time polymerase chain reactions performed on RNA isolated from native and epirubicin-resistant cell lines. Bar graphs indicate average quantitative means, while error bars represent standard error of the mean. *p* Values were calculated using unpaired *t* test. *ns* non-significant. **d** Immunoblotting of total H2A and H2B histone proteins in native and epirubicin-resistant cell lines. Glyceraldehyde-3-phosphate dehydrogenase (GAPDH) was used as a housekeeping control. **e** Reactome pathways significantly enriched within the module shown in (**b**)

Whole genome expression analysis revealed 209 genes in common, differentially expressed between all four pairs of native and epirubicin-resistant cell lines (Fig. 4a). Of these, 61 genes were regulated in the same direction in all four cell lines; of these, 26 genes were consistently upregulated and 35 were consistently downregulated (Additional file 1: Table S2 and Figure S3). These 61 genes were used to generate a gene interaction network and identify candidate pathways involved in epirubicin resistance. A minimal set of linker genes was used to connect the network. Identifying clustered genes within the network revealed four modules (Additional file 1: Figure S2); however, only modules 1 and 2 contained significantly enriched pathway annotations with a FDR less than 0.01. Module 1 contained three histone genes (*HIST1H2AC*, *HIST1H2BK* and *HIST1H2BD*) and several genes involved in RNA processing and mitosis (Fig. 4b). Importantly, all three histone genes were upregulated in all four cell lines and directly interconnected without linker genes. Within module 1, significantly enriched pathways included cell-cycle regulation (Fig. 4e), consistent with our results shown in Fig. 3. Module 2 contained three directly connected genes (*TACC3*, *AURKA* and *NFKBIA*) involved in aurora A kinase signalling. While *NFKBIA* was upregulated, *TACC3* and *AURKA* were downregulated.

We focused on histone-containing module 1 because all three histones were upregulated, tightly interconnected without linker genes and implicated in several molecular pathways. Elevated levels of all three histone transcripts were validated by qRT-PCR (Fig. 4c). Because antibodies specific to individual histone variants are not commercially available, we assessed protein expression using pan H2A and H2B antibodies. We observed no difference in the total H2A and H2B levels between resistant and native cell lines (Fig. 4d). Overall, our findings suggest that histone upregulation is a common event associated with epirubicin resistance in breast cancer cells and that histone-related pathways might be functional drivers of epirubicin resistance.

### Histone gene knockdown is insufficient to resensitise breast cancer cells to epirubicin

**Fig. 5:**
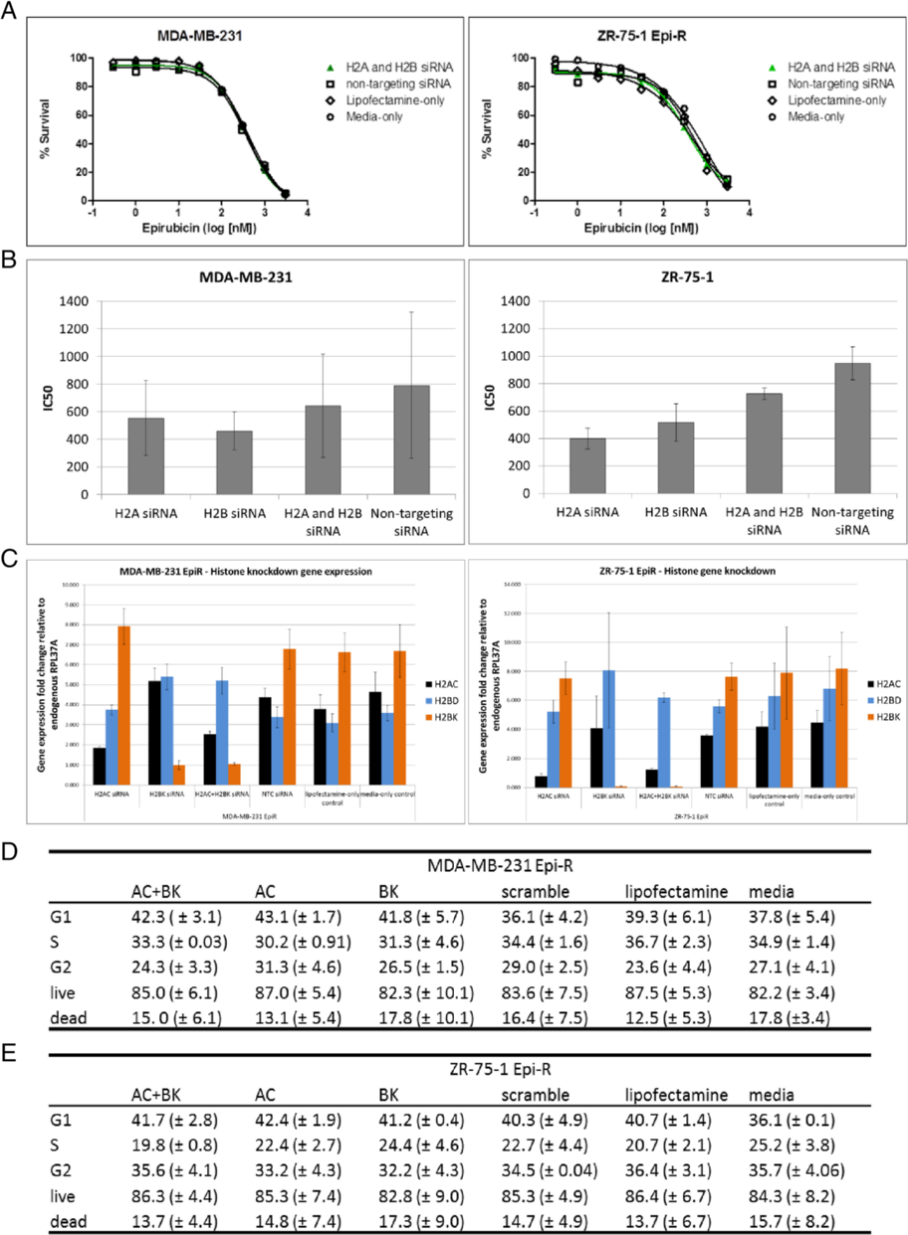
Histone gene knockdown is not sufficient to resensitise breast cancer cells to epirubicin. **a** A total of 7 × 10^4^ ZR75-1 epirubicin-resistant (EpiR) cells and MDA-MB-231 EpiR cells were transfected with 30 nM of each siRNA (Dharmacon; GE Healthcare, Lafayette, CO, USA) targeting *HIST1H2AC* and *HIST1H2BK* (individual knockdowns not shown for simplicity). Negative controls included media only, Lipofectamine only or mock transfection with non-targeting siRNA. Percentage gene expression knock-down is shown in Additional file 1: Table S3. **b** IC_50_ values were generated using non-linear regression analysis, and average values of two independent experiments are graphed. Error bars represent standard deviation. **c** Fold changes in gene expression of each histone variant relative to the housekeeping gene, *RPL37A*. **d-e** Histone knockdown effects on cell cycle and apoptosis for MDA-MB-231 and ZR-75-1. Tables show average percentages of cells in G1, S and G2 stages of cell cycle, and precent live versus dead cells in each experimental and control condition. Numbers in parenthesis indicate standard deviation between two experiments

We performed a series of gene knockdown experiments in MDA-MB-231- and ZR-75-1-resistant cells in which *HIST1H2AC*, *HIST1H2BK* or both were silenced before cells were exposed to epirubicin (Fig. 5a and b). A decrease in histone transcripts was confirmed by qRT-PCR (summarised in Additional file 1: Table S3). *HIST1H2BK*, rather than *HIST1H2BD*, was selected because high transcript levels of this variant were associated with poor survival of patients with breast cancer in our *in silico *analysis (data not shown; for online tool, see [21]). Interestingly, transient knockdown of either histone alone, or both, did not increase their sensitivity to epirubicin (Fig. 5a and data not shown), although epirubicin IC_50_ values were slightly reduced for ZR-75-1 upon histone knockdown (Fig. 5b). We further investigated whether the histone variant knockdown had an effect on cell cycle and apoptosis and found that neither process was severely affected (Fig. 5d and e). These results suggest that other histone variants might have compensated for the loss of *HIST1H2AC* and/or *HIST1H2BK* activity and that our efforts should be focused on targeting the function of the module rather than a few of its genes.

### Histone module is a clinical marker of anthracycline sensitivity

The prognostic significance of the 18-gene histone module was tested on the entire BR9601 clinical cohort, regardless of allocated adjuvant chemotherapy. High histone module expression was associated with reduced DRFS (HR 2.64, 95 % CI 1.7–4.09, *p* = 1.44 × 10^−5^), indicating that elevated histone module is prognostic of poor DRFS on chemotherapy.

**Fig. 6:**
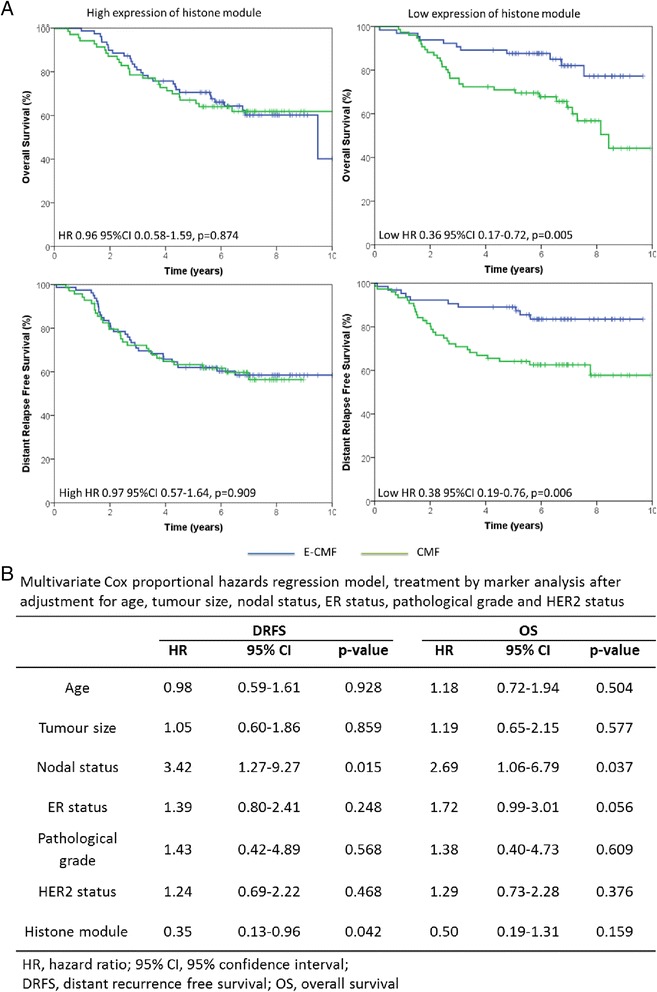
Histone module is a biological marker for anthracycline therapy. High expression and low expression of histone module were tested for association with distant recurrence-free survival (DRFS) and overall survival (OS) in the BR9601 trial, in which patients were treated with standard chemotherapy (CMF) or anthracycline-containing chemotherapy (E-CMF). **a** DRFS and OS for patients treated with E-CMF vs. CMF split into high or low histone gene expression groups. **b** Multivariate, treatment by marker analysis after adjustment for human epidermal growth factor receptor 2 (HER2) status, oestrogen receptor (ER) status, nodal status, grade and age. *HR* hazard ratio, *CI* confidence interval

Next, we analysed the differential effects of the histone module on breast cancer–specific overall survival (OS) and DRFS between patients in the BR9601 trial receiving an anthracycline (E-CMF) and those given CMF alone by assessing HRs and treatment by marker interactions. Patients whose tumours had low gene expression had increased OS (HR 0.38, 95 % CI 0.19–0.76, *p* = 0.005) when treated with E-CMF compared with patients treated with CMF alone. Conversely, there was no apparent differential benefit of E-CMF vs. CMF in patients with high histone module expression for OS (HR 0.97, 95 % CI 0.57–1.64, *p* = 0.91) (Fig. 6a). Similarly, patients whose tumours had low histone module expression had increased DRFS (HR 0.35, 95 % CI 0.17–0.73, *p* = 0.0048) when treated with E-CMF compared with patients treated with CMF alone (Fig. 6a). There was no apparent differential benefit of E-CMF vs. CMF in patients with high histone module expression for DRFS (HR 0.96, 95 % CI 0.58–1.59, *p* = 0.87). In multivariate analysis, after adjustment for HER2 status, nodal status, age, grade, size and ER status, the treatment by marker interaction showed no statistical difference for OS (HR 0.50, 95 % CI 0.19–1.31, *p* = 0.159). The likelihood of DRFS remained low among patients with low histone module gene expression compared with patients with high expression (HR 0.35, 95 % CI 0.13–0.96, *p* = 0.042) (Fig. 6b).

### HDACi induce cytotoxicity in epirubicin-resistant cells lines

**Fig. 7:**
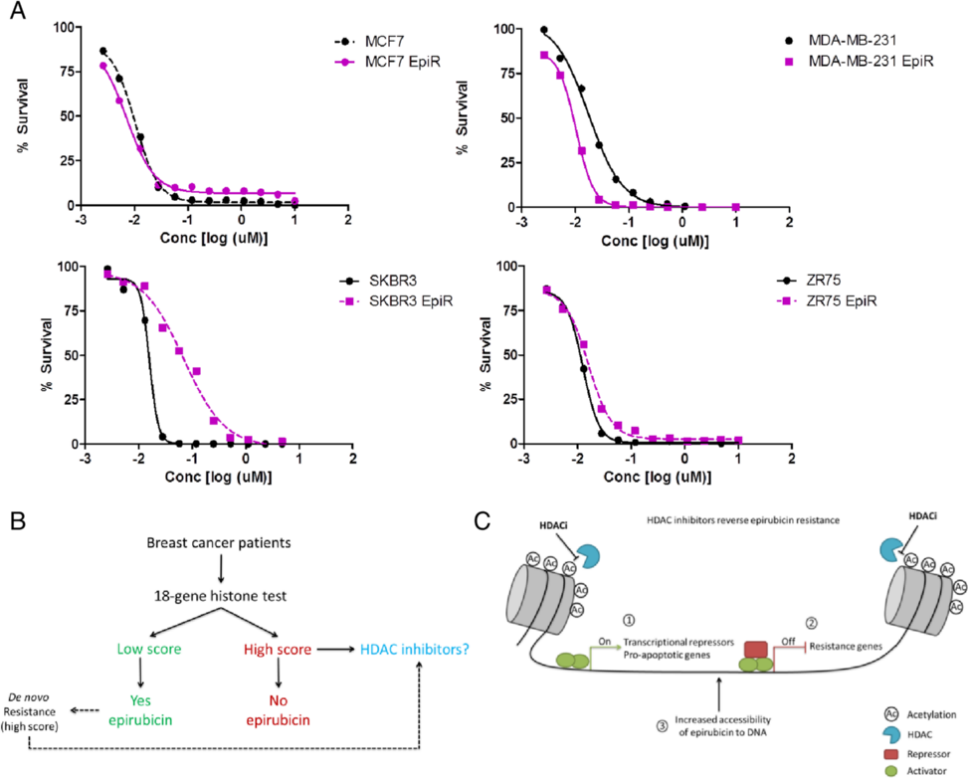
Histone deacetylase inhibitors (HDACi) induce cytotoxicity in epirubicin-resistant (EpiR) cell lines. **a** An example of the effect of panobinostat on all cell lines. Half-maximal inhibitory concentration values for panobinostat and the remaining HDACi are shown in Additional file 1: Table S4. **b** A schematic indicating that HDACi could offer a viable treatment option for patients who do not respond to anthracyclines (high histone score). In addition, patients with low histone score who would initially benefit from epirubicin treatment may in due course develop de novo resistance, and, if diagnosed with “high histone score” at recurrence, may be offered HDACi as a subsequent treatment option. **c** Working models of molecular mechanisms involved in epirubicin resistance. There are three proposed mechanisms by which HDACi sensitise cells to epirubicin: (1) by transcriptional activation of repressors and pro-apoptotic genes, (2) by repression of resistance genes and (3) due to increased accessibility to DNA

Gene expression analysis identified the histone module as significantly altered and possibly functionally required for epirubicin resistance. Consequently, we tested whether alteration of histone activity would sensitise cells to epirubicin using HDACi, which reverse histone hypoacetylation and permit transcriptional activation. Twenty-four HDACi were tested against the native and epirubicin-resistant cell lines. For resistant cell lines, all inhibitors were tested in the presence of selection doses of epirubicin. Positive hits were defined as compounds that exhibited cytotoxicity in at least 50 % of the population and had an IC_50_ less than 5 μM in all eight cell lines. We found that 14 HDACi were cytotoxic to all native and epirubicin-resistant cells lines (Additional file 1: Table S4). Importantly, three of four resistant cell lines were more sensitive to epirubicin than to native cells when several HDACi were supplied. The results provided in Fig. 7a show the effects of one HDACi, panobinostat, on all four cells lines. Interestingly, epirubicin-resistant MDA-MB-231 cells, but not MCF7 or ZR-75-1 cells, were more sensitive to panobinostat than native cells. In contrast, epirubicin-resistant SKBR3 cells were less sensitive to the HDACi than native cells, possibly due to the upregulation of efflux pumps (Fig. 2). Furthermore, because HDACi target different HDACs and none of the HDACi ubiquitously resensitised all four resistant cell lines (Additional file 1: Table S4), it appears that different classes of HDACs are involved in anthracycline resistance, possibly in a breast cancer subtype-specific manner. Collectively, our data reveal a previously unrecognised role of histones and suggests that H2A and H2B histones are involved in clinical anthracycline resistance.

## Discussion

Anthracycline resistance is a major obstacle to the effective treatment of women with breast cancer. Although various mechanisms may contribute to anthracycline resistance, including activation of drug transporters, reduced activity of Topo IIα and inhibition of apoptosis, the majority of the molecular mechanisms involved in clinical drug resistance remain unknown. Using a panel of cell lines representative of the major molecular subtypes of breast cancer, we have shown that deregulation of histones involved in chromosome maintenance, epigenetic pathways, cell division and gene regulation is observed consistently in epirubicin-resistant cell lines. This observation was then validated clinically in the BR9601 adjuvant clinical trial.

Histone *H2A* and *H2B* variants are emerging as mediators of drug sensitivity and resistance in cancer [22, 23]. We have shown that the dysregulation of histones is associated with increased cell-cycle progression, specifically the release of a G_2_/M cell-cycle block in the presence of epirubicin, and with a reduction in apoptotic cell death. Interestingly, transcriptional knockdown of the two histone variants contributing to the dysregulation signature did not completely sensitise cells to anthracycline, possibly for a few reasons. First, although the transcript levels were reduced by 6–53 %, it is possible that the protein levels remained unchanged during our experimental window. We were not able to assess protein expression of each specific variant, because antibodies are not yet commercially available. Second, even if the protein levels were sufficiently diminished, it is still possible that other histone variants functionally substituted for HIST1H2AC and HIST1H2BK because there are 9 *H2A* and 11 *H2B* non-allelic histone variants [24]. Third, the module contains 16 other genes that perform together with the histone genes in this functional module. This notion is shifting away from the previous efforts that were focused on discovering single genes as biomarkers by using fold-change differences in gene expression as the means of selecting promising biomarker candidates. Instead, the FI network approach relies on the strength of the gene-to-gene interactions and is based on how closely the genes are functionally related. This entire module was identified to be a predictive biomarker of anthracycline benefit, which allowed us to focus our efforts on identifying a drug that could target the function of an entire module rather than one of its components. Indeed, using a small-molecule inhibitor screen, we have shown that drugs directly targeting histone function (HDACi as well as cell-cycle inhibitors; data not shown) are cytotoxic to epirubicin-resistant cells and could be considered as an alternative treatment option for patients who do not respond to epirubicin (Fig. 7b). Collectively, these data suggest that modification of histone-regulated pathways represents a key “druggable” target in patients with epirubicin-resistant breast cancers.

Epirubicin-resistant cell lines were generated by exposing native, non-resistant cell lines to increasing concentrations of epirubicin. Interestingly, only a single cell line, SKBR3, upregulated drug transporters, and this was associated with cross-resistance to taxanes. Previously, Hembruff et al. [25] developed epirubicin-resistant MCF7 cells and established that a specific selection dose must be surpassed to activate drug transporters. For MCF7, this critical threshold concentration was around 30 nM [19]. Although this concentration is identical to the selection dose of our resistant MCF7 cells, MDR was not upregulated, suggesting a stochastic nature of molecular events that take place en route to drug resistance. Importantly, it indicates that there exists a previously unappreciated MDR-independent mechanism of resistance that should be evaluated for clinical relevance.

Our study revealed that one of those mechanisms involves upregulation of *H2A* and *H2B* genes and several pathways, including epigenetic and cell-cycle pathways. H2A and H2B histones form octamers with H3 and H4 histones, which participate in packaging of DNA into nucleosomes [26]. These histones are replication-dependent and cell-cycle–regulated, increasing 35-fold in S phase during DNA replication [27]. Thus, elevated histone transcript levels may be a consequence of a stalled cell cycle as cells struggle to repair epirubicin-induced DNA damage. However, because resistant cells did not stall, we eliminated the possibility that upregulated histone transcripts were a mere reflection of accumulated mRNA.

An alternative explanation, supported by the ability of HDACi to sensitise resistant cells to epirubicin, is that upregulation of histones contributed to (1) activation of resistance pathways, (2) silencing of molecular pathways that sensitise cells to anthracyclines, and/or (3) decreased accessibility of epirubicin to DNA. H3 and H4 histone modification patterns strongly associate with either active or repressed gene transcriptional status. Current understanding of H2A and H2B histone modifications is based on studies in yeast and few tumour cell lines; nonetheless, a few important features of H2A and H2B histone modifications have been revealed. First, modified sites are acetylated, phosphorylated and ubiquitinated, but not methylated [28–30], a modification most commonly observed with H3 and H4 histones. This highlights the appropriate use of HDACi in our study and their potency due to numerous acetylation sites, although this does not eliminate the possibility that the inhibitors were acting on H3 and H4 histones as well. Because acetylated sites on H2A and H2B are associated with transcriptional activation [28, 29], modifying the acetylation pattern may have activated transcriptional repressors and pro-apoptotic genes outlined in our model (Fig. 7c, point 1, *left*). Second, the N-terminal ends of H2A and H2B histones possess a repression domain that inactivates gene transcription in approximately 10 % of the yeast genome [28, 29], suggesting that these domains could have collaborated with acetylation patterns induced by HDACi to repress genes involved in resistance, such as those involved in cell cycle or apoptosis (Fig. 7c, point 2, *centre*). Third, our model also recognises that resistance might have been reversed by an increased accessibility of epirubicin to DNA (Fig. 7c, point 3, *right*).

Interestingly, although the cell lines were resistant, increasing epirubicin concentrations were ultimately cytotoxic to the cells, being indicative of partial drug resistance. This could be a consequence of dynamic changes in the population that is heterogeneous in terms of mutations [31] and the ability to use different resistance mechanisms at increasing concentrations of epirubicin until toxic levels are reached. Alternatively, a non-mutation–driven model might have contributed to the partial resistance to epirubicin; that is, because the rate of cell kill is proportional to the rate of tumour growth [32, 33], the effectiveness of epirubicin might have depended on the proportion of cells that were actively dividing vs. those that were in G_0_ phase and unresponsive to the drug treatment until they entered the G_1_ phase of the cell cycle [31].

Regel et al. [34] showed that the HDACi panobinostat sensitises gastric cancer cells to anthracyclines. Our findings are consistent with those of their study and show that multiple HDACi reverse anthracycline resistance in breast cancer cells. This is an important finding because many of the pharmacological inhibitors tested in our study are in use either as single agents or as combination therapies in phase II/III clinical trials [35–37]. HDACi currently in clinical trials include panobinostat, quisinostat, givinostat, abexinostat, pracinostat, belinostat and mocetinostat (Additional file 1: Table S4). Because anthracycline resistance may lead to cross-resistance to taxanes [2, 38], as it did in one of our resistant cell lines, it may be that taxanes, not anthracyclines, should be used in first-line treatment [39]. Furthermore, the patients in this study received polychemotherapy as part of their standard of care and, as was appropriate at the time, received either CMF (standard of care at the time) or anthracyclines (experimental at the time). Given the focus of our research on identifying markers of anthracycline benefit, this trial design satisfies the requirement of Simon et al. for a biomarker validation study [40]; however, further research in the context of taxane-based chemotherapy would be of value. As cancer cells could acquire resistance to HDACi [36], sequential therapy involving HDACi, taxanes and anthracyclines will be an important aspect of clinical trial design and medical practice.

## Conclusions

We have developed a relevant model for studying clinical resistance and a model that could be used for developing and testing novel single or combination breast cancer therapies. Importantly, we have identified novel pathways containing histone *H2A* and *H2B* genes as a mechanism of drug resistance across a spectrum of breast cancer cell lines and validated this finding in the BR9601 adjuvant clinical trial cohort. Although further validation studies are necessary, the use of HDACi holds promise for patients whose breast cancers do not respond to anthracycline-containing chemotherapy.

## Additional files

Additional file 1: Figure S1. Clinical trial BR9601 information. (**a**) Schematic representation. (**b**) Patient information. **Figure S2.** Entire FI network from 61 consistently changing genes. *Red circles* = upregulated genes; *green circles* = downregulated genes; *diamonds* = linker genes. **Figure S3.** Heat maps of probes for the 61 consistently changing genes. Rows are labelled with gene symbol and microarray probe IDs. (**a**) Raw expression values. (**b**) Row scaled expression values. **Figure S4.** Combination of pre-processing methods. The most optimal method selected was at the top(high-rank). **Figure S5.** Sample-by-gene heat map. Rows represent patients and columns represent genes. Both were clustered using a ward clustering algorithm. **Figure S6.** FI network generated from the histone module. *Circles* = genes within the module, *diamonds* = linker genes. **Figure S7.** Multiplot showing scaled mRNA abundance levels for each gene. A treatment-by-marker interaction Cox proportional hazards model was fit for each gene, and results are visualized on the *right* with the *squares* representing the hazard ratios (HRs) and the ends of the segments representing the 95 % confidence intervals in log_2_ scale. Patients are sorted by DRFS events on the *x*-axis and genes by decreasing log_2_ HR on the *y*-axis. **Figure S8.** Flow cytometric analysis of HER2 in MCF7 cells. *Yellow dotted line* represents HER2- cell line (MDA-MB-231), while *purple dotted line* shows HER2 amplified cell line (SKBR3). *Green* and *blue solid lines* represent native and epirubicin-resistant MCF7 cell lines, respectively, and show they are negative for HER2 cell surface expression. **Table S1.** Doubling times (in hours) of breast cancer cell lines. **Table S2** List of 61 common genes with consistent differential expression across all four cell lines. **Table S3.** Percentage reduction in gene expression compared with non-targeting siRNA control. **Table S4.** Drugs targeting epirubicin-resistant breast cancer cells. **Table S5.** List of primary antibodies. **Table S6.** List of histone module genes in the NanoString CodeSet. (PDF 1653 kb) Additional file 2:
**Description of mRNA abundance data processing, module dysregulation score (MDS) and survival modelling.** (PDF 360 kb)

## Abbreviations

CCK-8: Cell Counting Kit-8
CI: confidence interval
CMF: cyclophosphamide, methotrexate, 5-fluorouracil
DMSO: dimethyl sulphoxide
DRFS: distant relapse-free survival
E-CMF: epirubicin-cyclophosphamide, methotrexate, 5-fluorouracil
EGFR: epidermal growth factor receptor
EpiR: epirubicin-resistant cells
ER: oestrogen receptor
FDR: false discovery rate
FI: Cytoscape Reactome Functional Interaction plugin in Cytoscape 2.8.3
GAPDH: glyceraldehyde-3-phosphate dehydrogenase
HDAC: histone deacetylase
HDACi: histone deacetylase inhibitor
HER2: human epidermal growth factor receptor 2
HR: hazard ratio
IC_50_: half-maximal inhibitory concentration
MDR: multidrug resistance
mRNA: messenger RNA
OS: overall survival
PR: progesterone receptor
qRT-PCR: quantitative real-time polymerase chain reaction
RIPA: radioimmunoprecipitation assay
RT-PCR: real-time polymerase chain reaction
siRNA: small interfering RNA
Topo II: type II topoisomerase
WCL: whole-cell lysates
