# Supplementary material for: Downregulation of histone H2A and H2B pathways is associated with anthracycline sensitivity in breast cancer
Source: Breast Cancer Res. 2016 Feb 6;18:16. doi: 10.1186/s13058-016-0676-6 (PMC4744406; doi:10.1186/s13058-016-0676-6)
Supplement: Additional file 1: Figure S1. — Clinical trial BR9601 information. (a) Schematic representation. (b) Patient information. Figure S2. Entire FI network from 61 consistently changing genes. Red circles = upregulated genes; green circles = downregulated genes; diamonds = linker genes. Figure S3. Heat maps of probes for the 61 consistently changing genes. Rows are labelled with gene symbol and microarray probe IDs. (a) Raw expression values. (b) Row scaled expression values. Figure S4. Combination of pre-processing methods. The most optimal method selected was at the top(high-rank). Figure S5. Sample-by-gene heat map. Rows represent patients and columns represent genes. Both were clustered using a ward clustering algorithm. Figure S6. FI network generated from the histone module. Circles = genes within the module, diamonds = linker genes. Figure S7. Multiplot showing scaled mRNA abundance levels for each gene. A treatment-by-marker interaction Cox proportional hazards model was fit for each gene, and results are visualized on the right with the squares representing the hazard ratios (HRs) and the ends of the segments representing the 95 % confidence intervals in log2 scale. Patients are sorted by DRFS events on the x-axis and genes by decreasing log2 HR on the y-axis. Figure S8. Flow cytometric analysis of HER2 in MCF7 cells. Yellow dotted line represents HER2- cell line (MDA-MB-231), while purple dotted line shows HER2 amplified cell line (SKBR3). Green and blue solid lines represent native and epirubicin-resistant MCF7 cell lines, respectively, and show they are negative for HER2 cell surface expression. Table S1. Doubling times (in hours) of breast cancer cell lines. Table S2 List of 61 common genes with consistent differential expression across all four cell lines. Table S3. Percentage reduction in gene expression compared with non-targeting siRNA control. Table S4. Drugs targeting epirubicin-resistant breast cancer cells. Table S5. List of primary antibodies. Table S6. List of histone module gene [file 13058_2016_676_MOESM1_ESM.pdf]

A

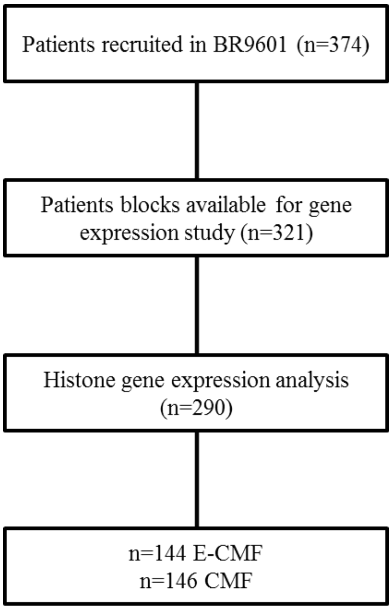

B

	Clinical trial	Histone analysis
Number	374	290
Age<br>Mean (range)	50.6<br>(22.7-76.0)	50.6<br>(26-76)
Treatment<br>E-CMF<br>CMF	 183 (48.9%)<br>191 (51.1%)	 144 (49.7%)<br>146 (50.3%)
Size<br><2.0 cm<br>>2.0 cm<br>Missing	 123 (32.9%)<br>251 (67.1%)	 83 (29.5%)<br>198 (70.5%)<br>9
Nodes<br>0<br>1-3<br>≥4<br>Missing	 48 (12.8%)<br>214 (57.3%)<br>112 (29.9%)	 42 (14.7%)<br>158 (55.5%)<br>85 (29.8%)<br>5
Grade<br>1<br>2<br>3<br>Unknown	 22 (6.1%)<br>126 (35.2%)<br>210 (58.7%)<br>16	 21 (7.3%)<br>93 (32.3%)<br>174 (60.4%)<br>2
ER Status<br>Positive<br>Negative<br>Unknown	 202 (62.9%)<br>119 (37.1%)<br>53	 159 (62.6%)<br>95 (37.4%)<br>36

Supplementary Figure 1: Clinical trial BR9601 information. A) Schematic representation of the patient samples available for analysis. B) Patient information available for the histone analysis.

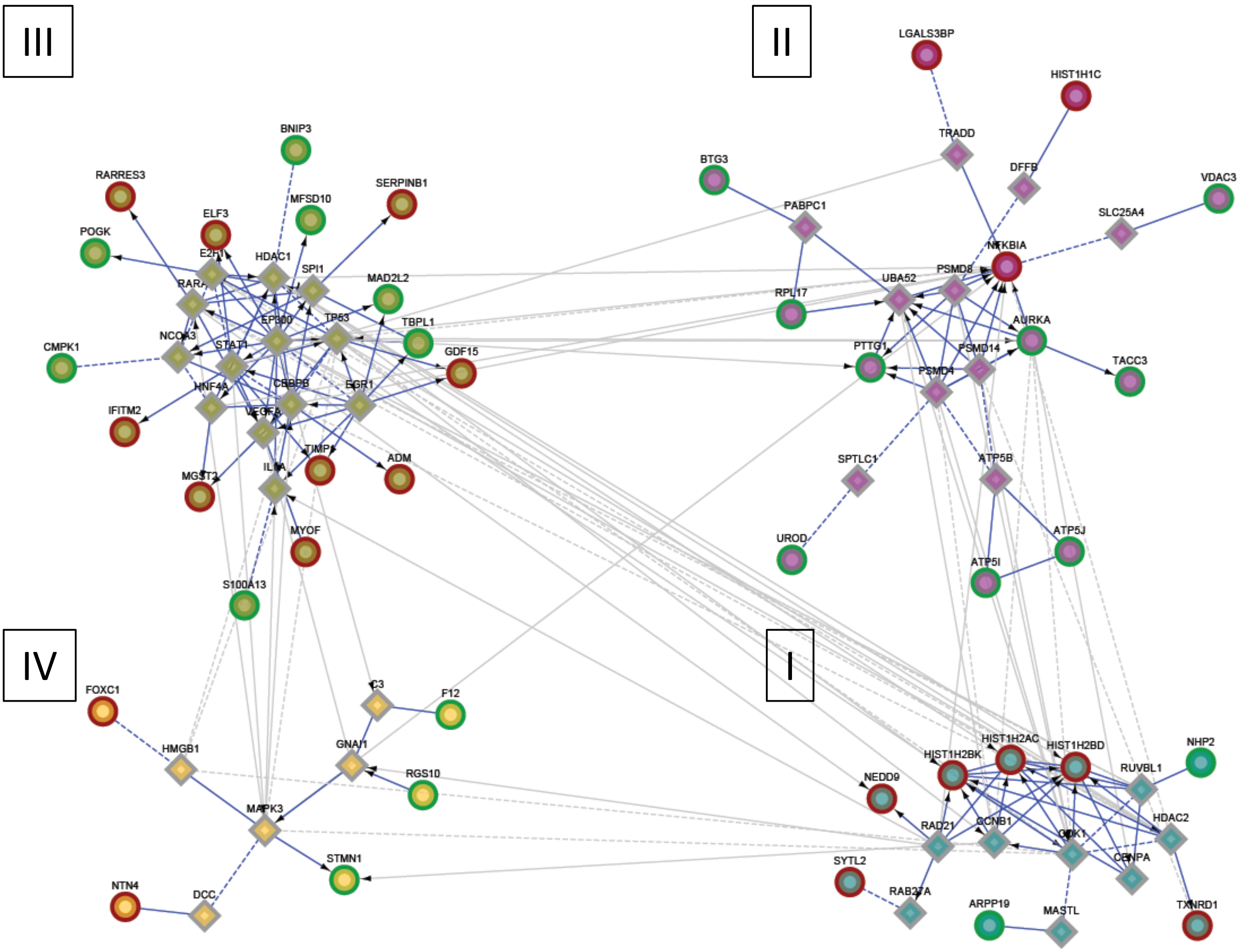

Supplementary Figure 2: Entire Functional Interaction network from 61 consistently changing genes. Red circles = upregulated genes ; green circles = downregulated genes; diamonds = linker genes.

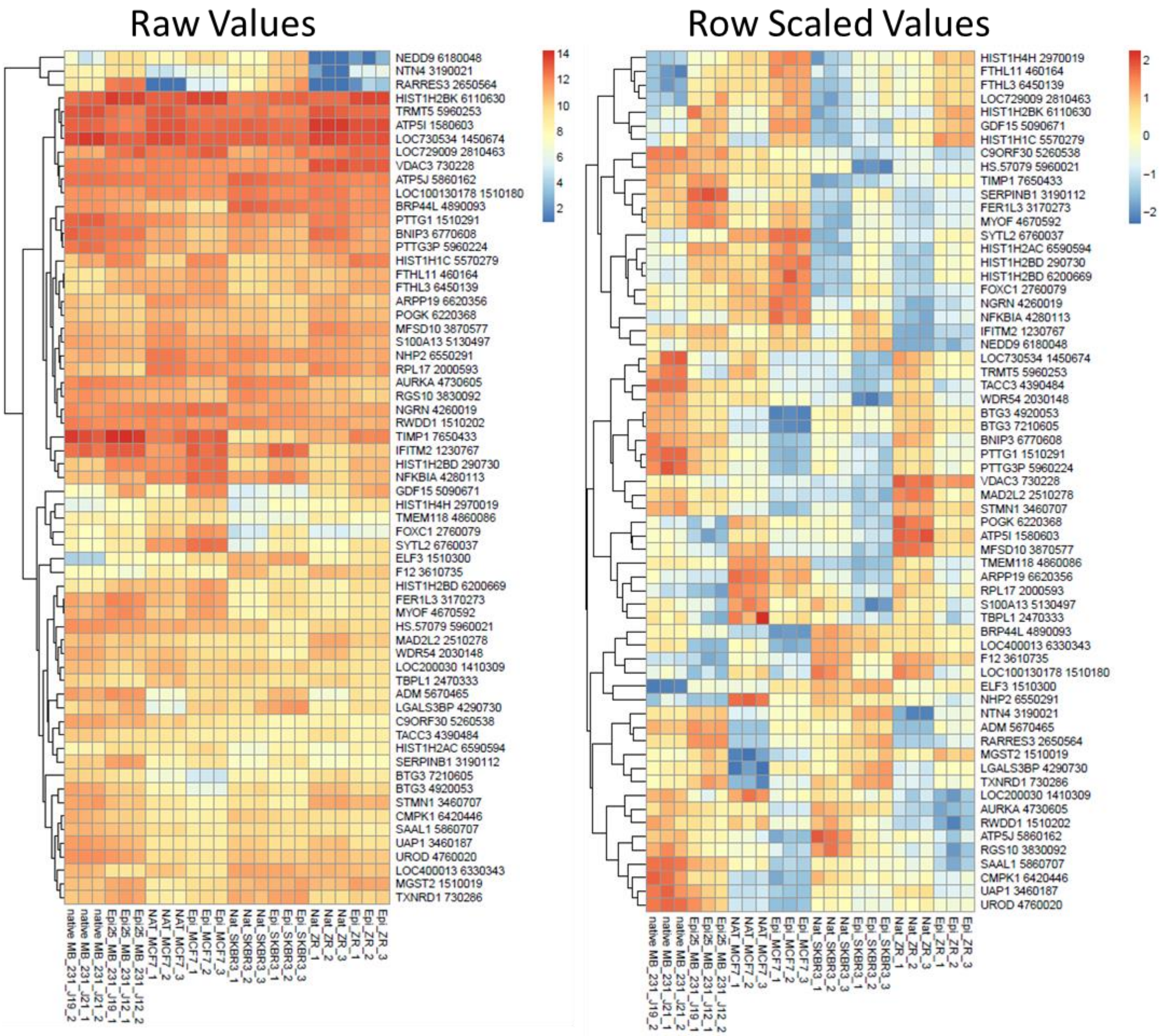

Supplementary Figure 3: Heatmaps of probes for the 61 consistently changing genes in four breast cancer cell lines. Rows labeled with gene symbol and microarray probe IDs. A) Raw expression values. B) Row scaled expression values.

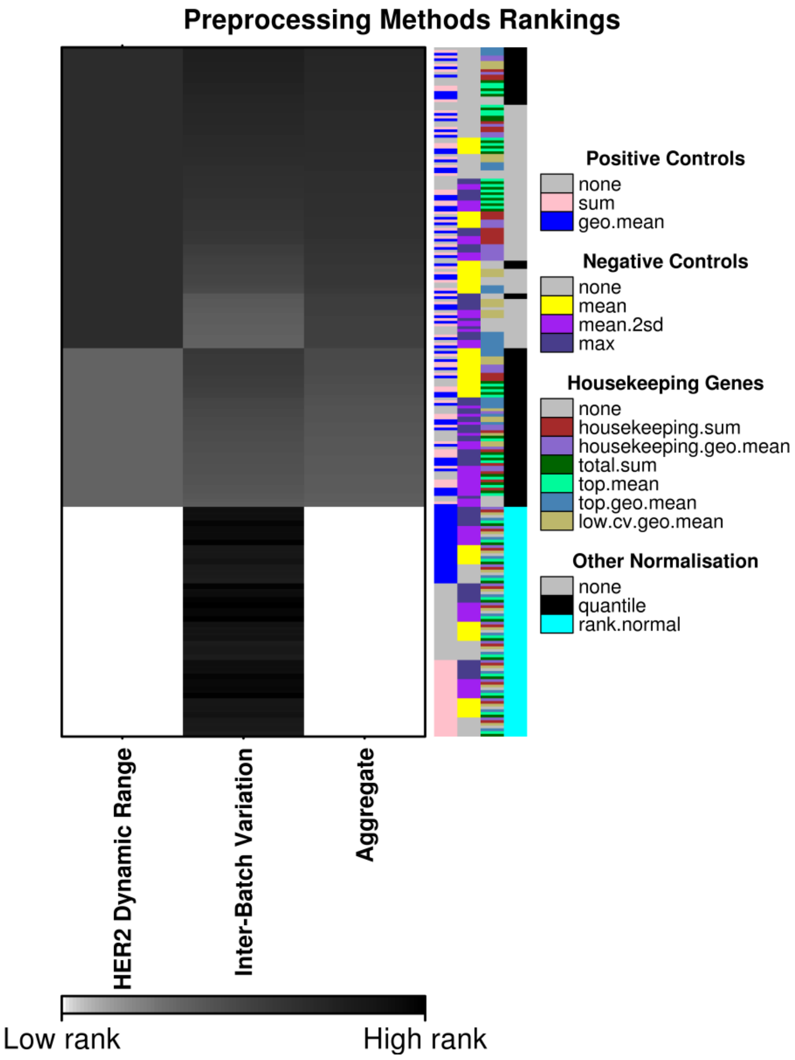

Supplementary Figure 4: Combination of pre-processing methods. The most optimal method selected was at the top, indicated by the black colour (high-rank).

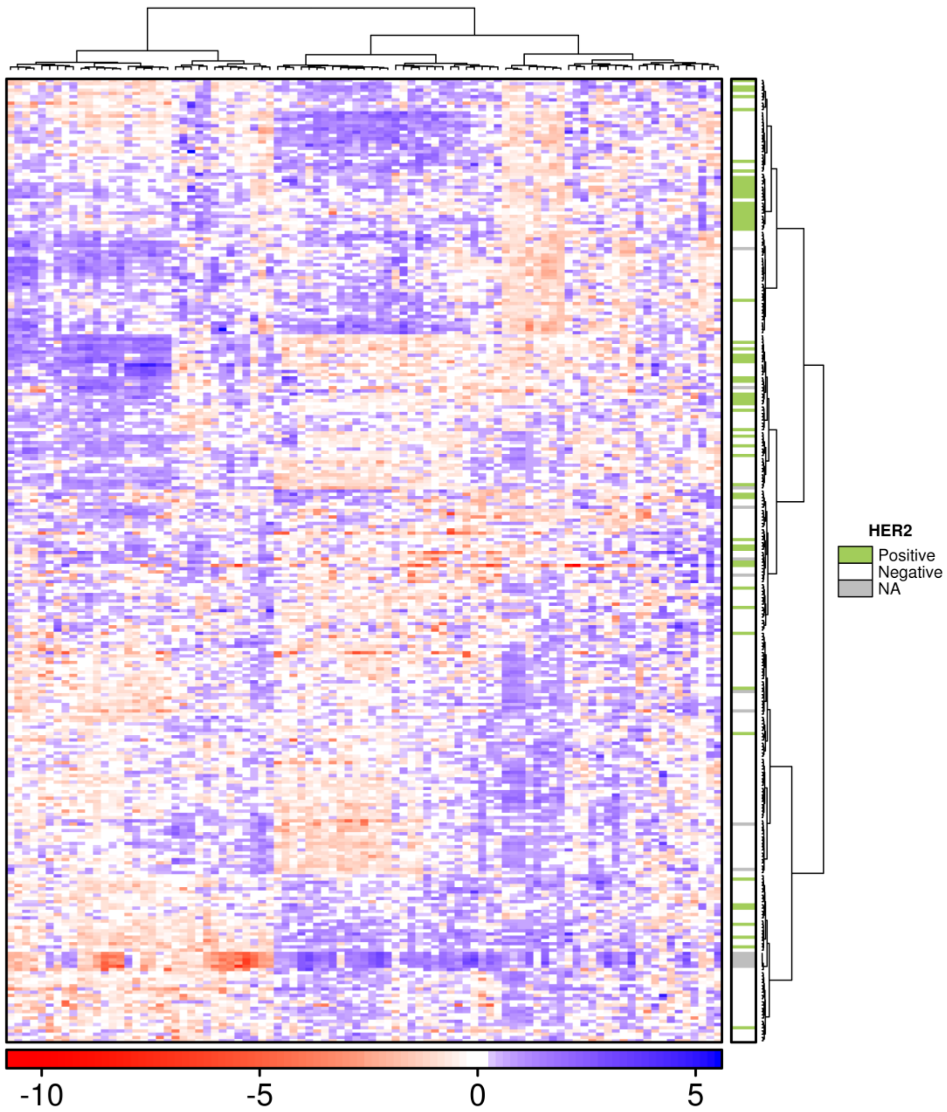

Supplementary Figure 5: Sample by gene heatmap. Rows represent patients and columns represent genes. Patients and genes are clustered using ward clustering algorithm.

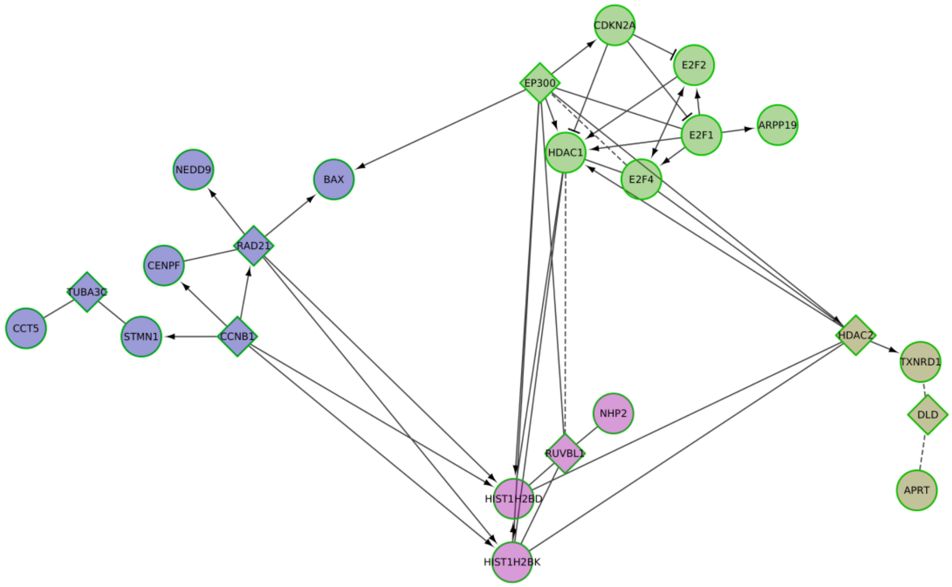

Supplementary Figure 6: Functional Interaction network generated from the histone module. Circles = genes within the module, diamonds = linker genes.

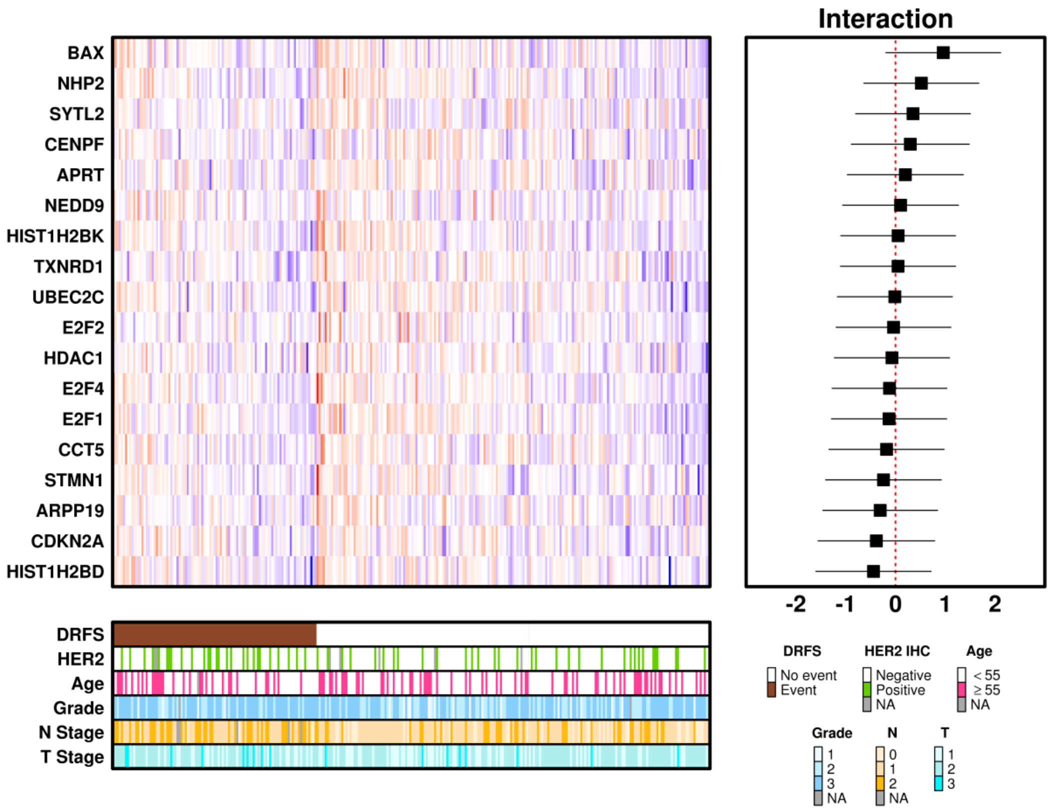

Supplementary Figure 7: Multiplot showing scaled mRNA abundance levels for each histone gene. A treatment-by-marker interaction Cox proportional hazards model was fit for each gene and results were visualized on the right with the squares representing the hazard ratios (HR) and the ends of the segments representing the 95% confidence intervals in log2 scale. Patients were sorted by DRFS events on the x-axis and genes by decreasing log2 HR on the y-axis.

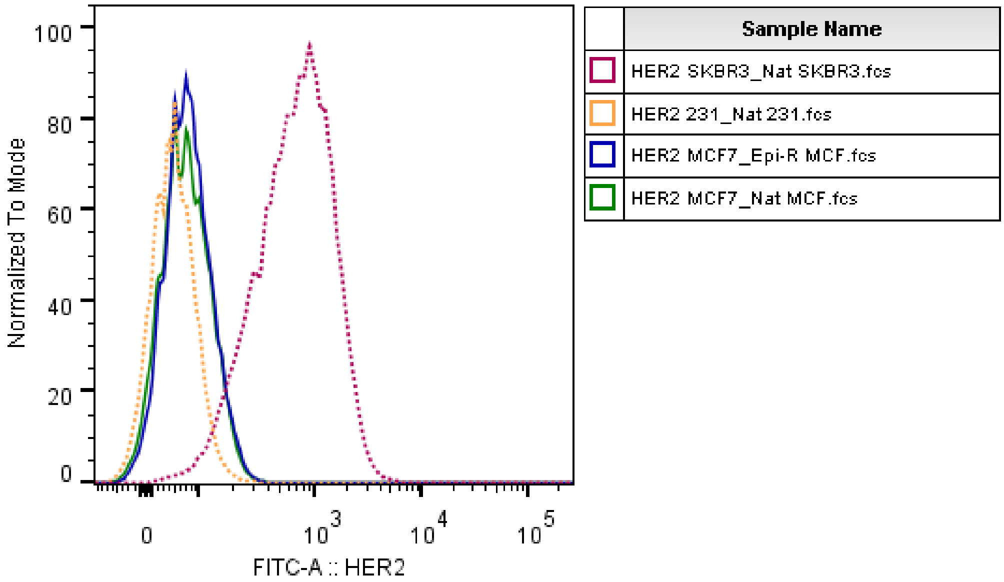

Supplementary Figure 8: Flow cytometric analysis of HER2 expression in MCF7 cells. Cells were gated by forward and side scatter. Yellow dotted line represents HER2 negative cell line (MDA-MB-231), while purple dotted line shows HER2 amplified cell line (SKBR3). Green and blue solid lines represent native and epirubicin-resistant MCF7 cell lines, respectively and show that they are negative for HER2 cell surface expression.

Supplementary Table S1: Doubling times (hours) of breast cancer cell lines

MDA-MB-231	- epirubicin	+ 25nM epirubicin
Native	25 (1.2)	70 (17.8)
25nM-Resistant	40 (4.2)	43 (3.0)
MCF7	- epirubicin	+ 30nM epirubicin
Native	29 (1.9)	74 (17.2)
30nM-Resistant	43 (4.1)	37 (4.7)
SKBR3	- epirubicin	+ 15nM epirubicin
Native	45 (3.2)	57 (6.6)
15nM-Resistant	63 (2.0)	66 (9.2)*
ZR-75-1	- epirubicin	+ 10nM epirubicin
Native	50 (8.1)	95 (14.2)
10nM-Resistant	72 (15.9)	67 (4.0)

Data is based on three independent experiments and shows standard deviation in parentheses. * Indicates data based on two experiments.

Supplementary Table S2: List of 61 common genes consistently differential across all four cell lines

ID	Symbol	Direction	GeomMe:	MB231_Log2FoldChange	MB231_AdjustedP	MCF7_Log2FoldChange	MCF7_AdjustedP	SKBR3_Log2FoldChange	SKBR3_AdjustedP	ZR_Log2FoldChange
2650564	RARRES3	Up	3.148596	3.653681782	3.63673E-06	4.447199806	4.25784E-07	2.768671149	9.64747E-05	2.184635223
6180048	NEDD9	Up	2.089221	3.741062631	7.0176E-07	1.58242038	0.007340759	2.850554148	2.09226E-05	1.128993412
5090671	GDF15	Up	2.032681	3.636941494	7.16273E-09	3.804855045	6.8255E-09	0.768664332	0.048864479	1.604966347
3190021	NTN4	Up	1.935478	1.241937436	0.001851238	1.556571883	0.000373031	1.678774226	0.000119633	4.324057875
6760037	SYTL2	Up	1.52817	1.1652976	0.000296203	1.011969203	0.001651786	2.500169094	2.23612E-08	1.849749141
2970019	HIST1H4H	Up	1.516285	1.80342791	3.58277E-07	1.090361289	0.000225985	1.770171737	4.13823E-07	1.518585301
4290730	LGALS3BP	Up	1.481281	0.682098757	0.047006282	3.964546016	1.12565E-09	1.447192416	0.000270078	1.230220759
2760079	FOXC1	Up	1.455519	1.00420363	0.01123889	1.249522673	0.003629325	4.20717645	9.50527E-10	0.850186644
5670465	ADM	Up	1.387493	0.487034246	0.009289321	2.638929722	3.75702E-11	1.285988414	4.96652E-07	2.24232699
1510300	ELF3	Up	1.381065	3.98007561	2.15212E-14	2.091234261	1.12565E-09	0.431898221	0.022036181	1.012000235
290730	HIST1H2BD	Up	1.227323	1.732788352	5.70725E-11	0.851313553	2.41395E-06	1.189402023	1.47803E-08	1.293217591
6590594	HIST1H2AC	Up	1.225358	1.403406756	1.31934E-07	0.880535196	7.12926E-05	1.478714456	5.6782E-08	1.233780479
6200669	HIST1H2BD	Up	1.185078	1.617462726	3.23951E-08	0.733181549	0.000702515	1.253651241	9.9673E-07	1.326676757
3190112	SERPINB1	Up	1.060114	1.204186672	1.70104E-07	1.165070852	4.04854E-07	1.792280786	3.93893E-10	0.502294954
5570279	HIST1H1C	Up	1.023357	1.027453541	0.000193176	1.856791331	2.39596E-07	0.528988612	0.032349843	1.086769647
4670592	MYOF	Up	0.911692	0.777614107	8.6546E-05	1.094263174	2.23886E-06	1.477305039	2.15583E-08	0.549587044
1230767	IFITM2	Up	0.882348	0.438443858	0.04703333	1.239747977	1.64764E-05	1.681706166	1.94374E-07	0.663073931
3170273	FER1L3	Up	0.877948	0.804031756	9.7142E-05	0.80661503	0.000141542	1.634891191	9.81454E-09	0.56033448
7650433	TIMP1	Up	0.793399	0.595283591	0.019559902	0.545339978	0.040652461	0.96714379	0.0006404	1.262073349
2810463	LOC729009	Up	0.785447	1.539336581	9.8671E-07	0.505607019	0.035900992	0.883796598	0.000571234	0.553310958
6450139	FTHL3	Up	0.752148	1.565607318	7.05118E-09	0.350509492	0.041522483	0.923646946	8.82241E-06	0.631428946
6110630	HIST1H2BK	Up	0.711506	0.861411469	1.9905E-06	0.663913117	7.01773E-05	0.494068326	0.00096232	0.906997373
460164	FTHL11	Up	0.680375	1.356498845	1.0086E-06	0.466130773	0.029280065	0.587851408	0.005957661	0.576499038
730286	TXNRD1	Up	0.66486	0.828723276	0.000140939	1.032238709	1.88443E-05	0.47312385	0.015299184	0.482787056
4280113	NFKBIA	Up	0.603038	0.443885758	0.018039201	0.602469714	0.003460198	0.664679665	0.001174192	0.743979416
1510019	MGST2	Up	0.588832	0.690699027	0.000233532	0.888303429	2.37996E-05	0.356187337	0.035432188	0.550092348
4260019	NGRN	Up	0.285379	0.284273928	0.017492979	0.286069278	0.023278031	0.278236487	0.022885132	0.293131687
4920053	BTG3	Down	1.057953	-0.994500313	7.60336E-08	-2.052756871	2.71104E-12	-0.707227434	6.47272E-06	-0.867687244
2030148	WDR54	Down	0.937605	-0.697640144	4.99278E-05	-0.695295447	7.93894E-05	-1.225403064	3.19761E-08	-1.300169246
7210605	BTG3	Down	0.914882	-0.782708938	0.000186404	-1.882321708	4.30457E-09	-0.52243878	0.006715335	-0.910188702
3610735	F12	Down	0.821478	-0.668310659	0.001690483	-0.731457558	0.001186424	-1.698184733	3.15993E-08	-0.548568688
3460707	STMN1	Down	0.776332	-0.881249308	2.49829E-06	-1.06145713	3.28522E-07	-0.990321397	5.10112E-07	-0.392113403
6770608	BNIP3	Down	0.728782	-0.355313963	0.012030965	-0.896084604	2.45297E-06	-0.707030937	3.06142E-05	-1.253116563
5960224	PTTG3P	Down	0.638443	-0.594570863	0.000701649	-0.713277749	0.000185456	-0.622173213	0.000517861	-0.62967301
1510291	PTTG1	Down	0.608276	-0.586131618	0.000147683	-0.878266532	1.99227E-06	-0.459314456	0.001625214	-0.578990073
3460187	UAP1	Down	0.605505	-0.609999942	7.63221E-05	-0.345489059	0.013592559	-0.530382744	0.000344145	-1.202591574
6330343	LOC400013	Down	0.6027	-1.497139045	2.5147E-10	-1.197130666	1.29734E-08	-0.255806511	0.04764818	-0.287799173
4390484	TACC3	Down	0.593789	-0.626709097	0.000129782	-0.657393468	0.00011824	-0.283671092	0.049577036	-1.063709121
2000593	RPL17	Down	0.577395	-0.588811817	0.005418746	-0.501744259	0.020892276	-0.70368187	0.001591708	-0.534634337
5260538	C9ORF30	Down	0.576402	-0.369061183	0.031841007	-0.719125251	0.000377899	-0.781155377	0.000113485	-0.532427825
3870577	MFSD10	Down	0.574913	-0.393204338	0.000955945	-1.018383073	1.6896E-08	-0.445862974	0.000332799	-0.611895834
2470333	TBPL1	Down	0.569527	-0.790059852	0.000223837	-0.516164639	0.010887416	-0.392221311	0.041959994	-0.657777182
4890093	BRP44L	Down	0.552822	-0.509230881	0.00013697	-0.918490682	1.64274E-07	-0.472373824	0.000320624	-0.422733179
3830092	RGS10	Down	0.551694	-0.599276133	0.001336879	-0.482359577	0.009910653	-0.633949005	0.000933824	-0.505522085
5860707	SAAL1	Down	0.543692	-0.441887147	0.001870805	-0.905719329	1.26247E-06	-0.301083107	0.027747176	-0.725135442
5130497	S100A13	Down	0.537715	-0.3731618	0.022268267	-0.532321743	0.003150339	-1.025263414	2.1919E-06	-0.410489866
2510278	MAD2L2	Down	0.518602	-0.314700606	0.022350536	-0.720882199	3.15681E-05	-0.3321464	0.019159199	-0.959945984
4860086	TMEM118	Down	0.510012	-0.431073739	0.00618594	-0.357621648	0.026680875	-0.32636258	0.036611528	-1.344764073
5960253	TRMT5	Down	0.496152	-0.864734207	6.53683E-07	-0.668965367	2.47573E-05	-0.279863322	0.028979187	-0.374305702
1410309	LOC200030	Down	0.478234	-0.681366125	0.000102772	-0.79004931	3.01351E-05	-0.312093958	0.0424021	-0.311344924
5960021	HS.57079	Down	0.471627	-0.300585974	0.014673571	-0.285037365	0.02698542	-2.070268939	8.65337E-13	-0.278931657
6620356	ARPP19	Down	0.442397	-0.321869883	0.014853613	-0.302991326	0.028320302	-0.528950623	0.000357123	-0.742545896
1580603	ATP5I	Down	0.442257	-0.627441919	2.37616E-06	-0.414234377	0.000397532	-0.296398647	0.005183639	-0.49659544
6420446	CMPK1	Down	0.441949	-0.750473772	3.8767E-05	-0.335178786	0.034964112	-0.456565239	0.004452136	-0.332179689
1510180	LOC100130178	Down	0.439856	-0.261347038	0.012650558	-0.491648564	9.41264E-05	-0.488091279	7.03782E-05	-0.596857031
1450674	LOC730534	Down	0.424832	-0.682052148	8.45419E-06	-0.525804233	0.000237981	-0.283328604	0.022515347	-0.320580401
4760020	UROD	Down	0.409187	-0.475845157	0.000536956	-0.438340937	0.001666825	-0.40946835	0.002206143	-0.328237685
4730605	AURKA	Down	0.408034	-0.281668226	0.042610896	-0.969683657	1.02828E-06	-0.291324158	0.042601704	-0.348371698
6550291	NHP2	Down	0.376524	-0.235508145	0.046581381	-0.930566464	2.15841E-07	-0.276751743	0.024576203	-0.33138139
1510202	RWDD1	Down	0.362583	-0.239714468	0.046435999	-0.469491498	0.000779742	-0.509735867	0.000256759	-0.301275026
6220368	POGK	Down	0.338358	-0.226483407	0.034515599	-0.310872883	0.00784246	-0.389836437	0.001038556	-0.477536144

Supplementary Table S3: Percent reduction in gene expression compared to non-targeting siRNA control

	MDA-MB-231 Epi-R		ZR-75-1 Epi-R	
	H2AC expression	H2BK expression	H2AC expression	H2BK expression
siH2AC	24.4 (±3.2)	-	27.5 (±0.16)	-
siH2BK	-	12.2 (±2.5)	-	5.7 (±1.44)
siH2BA and siH2BK	40.7 (±10.9)	12.2 (±3.7)	52.8 (±0.99)	7.9 (±2.25)

Supplementary Table S4: Drugs targeting epirubicin-resistant breast cancer cells

Drug status	Drug name	IC_{50} values (μM)							
		MCF7 Nat	MCF7 EpiR	231 Nat	231 EpiR	SKBR3 Nat	SKBR3 EpiR	ZR75 Nat	ZR75 EpiR
Phase III	Panobinostat (LBH-589)	0.01	0.01	0.02	0.01	0.02	0.07	0.01	0.02
Phase II	Quisinostat (JNJ-26481585)	0.01	0.01	0.01	0.01	0.01	0.22	0.01	0.01
Phase II	Givinostat (ITF2357)	0.10	0.08	0.26	0.16	0.22	2.74	0.17	0.18
Phase II	Abexinostat (PCI-24781)	0.11	0.09	0.27	0.12	0.21	2.25	0.14	0.16
Phase II	Pracinostat (SB939)	0.16	0.12	0.54	0.18	0.26	0.92	0.15	0.23
Phase II	Belinostat (PX-105684)	0.25	0.20	0.50	0.18	0.21	0.15	0.36	0.46
Phase II	Mocetinostat (MGCD0103)	0.32	0.41	0.85	0.43	1.00	3.69	0.35	0.43
Preclinical	Apicidin A (OSI-2040)	0.07	0.11	0.23	0.11	0.17	2.21	0.21	0.25
Preclinical	CAY10603 (ST-2-92)	0.61	0.38	1.27	0.82	0.44	1.03	0.98	0.75
Preclinical	Oxamflatin (107-0130)	0.62	0.25	0.59	0.29	1.28	0.69	0.68	1.20
Preclinical	Trichostatin A	1.18	0.50	0.33	0.15	1.52	1.24	1.83	2.28
Preclinical	Scriptaid	1.34	0.72	3.81	1.30	1.25	0.94	1.66	1.23
Tool compound	CBHA	1.18	3.58	2.39	1.75	1.45	1.03	2.94	2.25
Discontinued - Phase I	Dacinostat (LAQ824)	0.02	0.01	0.04	0.02	0.02	0.06	0.02	0.02

Supplementary Table S5: List of primary antibodies

Antibody	Vendor	Clone
anti-EGFR	Santa Cruz Biotech	A-10
anti-PR	Dako	PgR 636
anti-HER2	Cell Signaling Technology	Polyclonal (#2242)
anti-HER3	Dako	DAK-H3-IC
anti-ERα	Novocastra/Leica	ER 6F11
anti-MDR1	Santa Cruz Biotech	G-1
anti-TOPOIIα	Cell Signaling Technology	D10G9
anti-H2A	Cell Signaling Technology	Polyclonal (#2578)
anti-H2B	Cell Signaling Technology	53H3
anti-actin	Calbiochem	JLA20
anti-GAPDH	Cell Signaling Technology	D16H11

Supplementary Table S6: List of histone module genes in the Nanostring codeset

HIST1H2BK
HIST1H2BD
NEDD9
SYTL2
NHP2
ARPP19
TXNRD1
CENPF
STMN1
CCT5
APRT
UBEC2C
BAX
HDAC1
E2F1
E2F2
E2F4
CDKN2A
