# Supplementary material for: Downregulation of histone H2A and H2B pathways is associated with anthracycline sensitivity in breast cancer
Source: Breast Cancer Res. 2016 Feb 6;18:16. doi: 10.1186/s13058-016-0676-6 (PMC4744406; doi:10.1186/s13058-016-0676-6)
Supplement: Additional file 2: — Description of mRNA abundance data processing, module dysregulation score (MDS) and survival modelling. (PDF 360 kb) [file 13058_2016_676_MOESM2_ESM.pdf]

mRNA Abundance Data Processing

Raw mRNA abundance counts data were preprocessed using R package NanoStringNorm (v1.1.19). In total, 252 preprocessing schemes were assessed, including the use of six positive controls, eight negative controls and six housekeeping genes (TRFC, TBP, GUSB, TMED10, SF3A1, and PUM1) followed by global normalization (Supplementary Figure 4). We used two criteria to help identify the optimal preprocessing parameters as previously described (Haider S., Yao C. Q., Sabine V. S., Grzadkowski M., Starmans M. H. W., Wang J., Nguyen F., Moon N. C., Lin X., Drake C., Crozier C. A., Brookes C. L., van de Velde C. J. H., Hasenburg A., Kieback D. G., Markopoulos C. J., Dirix L. Y., Seynaeve C., Rea D. W., Kasprzyk A., Lambin P., Lio P., Bartlett J. M. S., Boutros P. C., unpublished data). First, each of the 252 combinations of preprocessing schemes was ranked based on their ability to maximize Euclidean distance of ERBB2 mRNA abundance levels between HER2-positive and HER2-negative patients. For robustness, the entire process was repeated for 1 million random subsets of HER2-positive and HER2-negative samples for each of the preprocessing schemes. Second, we included 5 replicates of an RNA pool extracted from randomly selected anonymized FFPE breast tumour samples; the rationale here was to assess each of the different preprocessing schemes for their inter-batch variation and rank them as previously described (Haider S., Yao C. Q., Sabine V. S., Grzadkowski M., Starmans M. H. W., Wang J., Nguyen F., Moon N. C., Lin X., Drake C., Crozier C. A., Brookes C. L., van de Velde C. J. H., Hasenburg A., Kieback D. G., Markopoulos C. J., Dirix L. Y., Seynaeve C., Rea D. W., Kasprzyk A., Lambin P., Lio P., Bartlett J. M. S., Boutros P. C., unpublished data). For this evaluation, a mixed effects linear model was used and residual estimate was used as a metric for inter-batch variation (R package: nlme v3.1-120). Lastly, we estimated the cumulative ranks using RankProduct (Breitling et al. 2004) based on the

two criteria and identified the optimal pre-processing scheme as using *geometric mean* derived from the top 75 expressing genes for sample content followed by *quantile normalisation* (Supplementary Figure 5). No samples were removed after QAQC. Six samples were run in duplicates, and their raw counts were averaged and subsequently treated as a single sample.

Module Dysregulation Score (MDS)

As previously described (Haider S., Yao C. Q., Sabine V. S., Grzadkowski M., Starmans M. H. W., Wang J., Nguyen F., Moon N. C., Lin X., Drake C., Crozier C. A., Brookes C. L., van de Velde C. J. H., Hasenburg A., Kieback D. G., Markopoulos C. J., Dirix L. Y., Seynaeve C., Rea D. W., Kasprzyk A., Lambin P., Lio P., Bartlett J. M. S., Boutros P. C., unpublished data), predefined functional modules were scored using a two-step process. First, weights (β) of all the genes were estimated by fitting a multivariate Cox proportional hazards model and were obtained from the treatment by marker interaction term (Training cohort only). Second, these weights were applied to scaled mRNA abundance profiles to estimate per-patient module dysregulation score using the following equation 1:

$$MDS = \sum_{i=1}^{n} \beta X_i \qquad (1)$$

where n represents the number of genes in a given module and X_i is the scaled (z-score) abundance of gene i. MDS was subsequently used in the multivariate Cox proportional hazards model alongside clinical covariates.

Survival Modelling

Using a stratified 5-fold cross validation approach, MDS profiles (equation 1) of patients within each training set were used to fit a univariate Cox proportional hazards model. The

parameters estimated by the univariate model were applied to patient-wise MDS in the testing set of each fold to generate per-patient risk scores. These continuous risk scores were dichotomized based on the median threshold derived from each training set, and the resulting dichotomized groups were evaluated through Kaplan-Meier analysis. Models were trained and validated using DRFS truncated to 10 years as an end-point.

Reference List

Breitling R, Armengaud P, Amtmann A and Herzyk P. (2004). *FEBS Lett,* **573,** 83-92.
